# Record‐Breaking Far‐Red Silicon Quantum Dots LEDs Enabled by Solvent Engineering: Toward Superseding Perovskite Quantum Dots

**DOI:** 10.1002/smsc.202400647

**Published:** 2025-04-16

**Authors:** Li Wang, Yuto Wada, Honoka Ueda, Temmaru Hirota, Kota Sumida, Yuito Oba, Ken‐ichi Saitow

**Affiliations:** ^1^ Department of Chemistry Graduate School of Advanced Science and Engineering Hiroshima University 1‐3‐1 Kagamiyama Higashi‐Hiroshima Hiroshima 739‐8526 Japan; ^2^ Department of Materials Science Natural Science Center for Basic Research and Development (N‐BARD) Hiroshima University 1‐3‐1 Kagamiyama Higashi‐Hiroshima Hiroshima 739‐8526 Japan

**Keywords:** far‐red luminances, light‐emitting diodes, non‐toxic quantum dots, silicons, solvent engineerings

## Abstract

Most quantum dots (QDs) contain either toxic elements, which are health and environmental hazards, or costly precious metals. In contrast, as nanocrystals consisting mainly of an earth‐abundant, light element, silicon QDs (SiQDs) have attracted attention as cost‐effective biomedical, display, and solid‐state lighting materials. However, unlike heavy‐metal or perovskite QDs, SiQDs have not yet been used to create high‐performance optoelectronics or long‐lifetime light‐emitting diodes (LEDs). Herein, the fabrication via solvent engineering of SiQD LEDs with record‐breaking external quantum efficiency (16.5%) and lifetimes up to 733 times longer than the previous record is reported. Furthermore, the far‐red (750 nm) luminance is comparable to that of state‐of‐the‐art perovskite QD LEDs. Dispersing the SiQDs in octane yields particularly efficient LEDs owing to negligible SiQD aggregation, and Joule heating minimization realizes long‐term stability (lifetime >200 h). Thus, solvent engineering is harnessed to break four QD LED performance records—for efficiency, luminance, voltage, and operational lifetime—using a more sustainable QD material, and the mechanisms underlying these performance improvements are unveiled. Thus, a new solvent‐engineering approach for developing efficient, stable, and sustainable far‐red SiQD LEDs, which are valuable light sources for applications including plant growth acceleration and photodynamic therapy, is highlighted.

## Graphical abstract

Solvent engineering is used to demonstrate that the light, nontoxic element Si, in the form of quantum dots (QDs), can function as the emissive material in light‐emitting diodes (LEDs) with performance rivalling that of toxic perovskite QD LEDs. These silicon QD (SiQD) LEDs set new records for efficiency, luminance, voltage, and lifetime, and how the choice of solvent impacts device performance is unveiled.

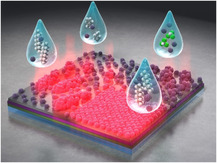

## 1 Introduction

The use of silicon as a semiconductor material in electronics and solar cells has revolutionized modern society, but this element has not yet been harnessed to realize commercial luminescent devices owing to the poor optical properties of its bulk crystalline form. Specifically, the photoluminescence quantum yield (PLQY) of silicon is typically low (≈0.01%)^[^ ^1^ ^]^ and its photoluminescence (PL) occurs at near‐infrared (IR) wavelengths. In contrast, since the discovery of light‐emitting porous silicon^[^ ^1^, ^2^, ^3^, ^4^, ^5^, ^6^ ^]^ by Canham, colloidal silicon quantum dots (SiQDs) have been extensively synthesized,^[^ ^7^, ^8^, ^9^, ^10^, ^11^, ^12^, ^13^, ^14^ ^]^ with PLQYs as high as 80–90%^[^ ^15^, ^16^, ^17^ ^]^ and PL wavelengths across the entire visible region thanks to quantum confinement and surface ligand effects.^[^ ^15^, ^18^, ^19^, ^20^, ^21^ ^]^

Silicon is a light element and the second most abundant element in the Earth's crust (28%) after oxygen (47%). It is particularly important as a possible means of resolving concerns about the toxic elements or precious metals found in most quantum dots (QDs), which are hazardous to human health and the environment and/or costly. Therefore, colloidal SiQDs have enormous potential as heavy metal–free (In‐, Cd‐, Pb‐, Zn‐, Cu‐, Ag‐, and Hg‐free), halogen‐free QDs to address the significant challenge of fabricating next‐generation QDs for displays, lighting, biomedical imaging,^[^ ^22^, ^23^ ^]^ luminescent solar concentrators,^[^ ^24^, ^25^ ^]^ and photon upconversion^[^ ^26^ ^]^ materials via solution processing. Moreover, the upcycling of discarded rice husks to prepare light‐emitting diodes (LEDs) based on SiQDs has been reported,^[^ ^27^ ^]^ demonstrating that these nanomaterials can be synthesized via eco‐friendly methods.

SiQD LEDs have attracted considerable attention, and the dependence of their optoelectronic performance on various design parameters—device structure,^[^ ^28^ ^]^ multilayer material band alignment,^[^ ^28^, ^29^, ^30^, ^31^ ^]^ layer thickness,^[^ ^16^, ^29^, ^32^ ^]^ SiQD size,^[^ ^31^, ^32^, ^33^, ^34^, ^35^ ^]^ surface ligand species,^[^ ^36^, ^37^ ^]^ and ligand coverage^[^ ^38^, ^39^ ^]^—has been investigated.^[^ ^15^ ^]^ However, these devices have three well‐known limitations that must be addressed. First, the electroluminescence (EL) wavelengths of almost all the SiQD LEDs reported thus far have been in the orange,^[^ ^27^, ^33^, ^40^ ^]^ red,^[^ ^27^, ^28^, ^29^, ^30^, ^32^, ^34^, ^35^, ^36^, ^37^, ^38^, ^39^, ^41^, ^42^, ^43^, ^44^ ^]^ or near‐IR^[^ ^31^, ^45^ ^]^ (to the best of our knowledge, there is just one exception among all the SiQD LEDs reported in the literature, an emission at white–blue wavelengths^[^ ^46^ ^]^). Second, thus far, SiQD LED performance has lagged behind heavy metal and perovskite QD LED performance. In the past decade, external quantum efficiencies (EQEs) in the range of 3–30% have been reported for QD LEDs made from heavy metals (CdSe [5–30%], InP [3–20%]), and perovskites (1–30%),^[^ ^47^, ^48^, ^49^ ^]^ whereas SiQD LED EQEs are typically ≈1%, although EQEs of 8–13% for SiQD LEDs with conventional structures^[^ ^31^, ^44^ ^]^ and 10–12% for those with inverted structures^[^ ^16^, ^42^ ^]^ have been reported. Further details of the current state‐of‐the art in terms of performance are presented in a recent review article on SiQD LED research.^[^ ^15^ ^]^ Third, the longest operation time (i.e., the greatest *T*_70_, the time required to reach 70% of the initial luminance) reported for an SiQD LED is 26 h,^[^ ^28^ ^]^ whereas *T*_70_ values of >6000 h (initial luminance: 985 cd m^−2^)^[^ ^50^ ^]^ have been estimated, based on experimental data, for heavy‐metal QD LEDs made using Cd, In, or perovskite QDs, and the record for the longest estimated *T*_70_ is 2 × 10^6^ h (initial luminance: 100 cd m^−2^).^[^ ^47^, ^51^ ^]^ The excellent performance of heavy‐metal and perovskite QD LEDs is in part owing simply to the large number of studies performed. Recent searches of the SciFinder scholar database found 2101 reports on Cd‐based QD LEDs, 618 on In‐based QD LEDs, and 659 on perovskite QD LEDs in the literature, whereas only 29 reports on SiQD LEDs are in existence as of January 2025. Thus, a greater focus on SiQD LED research is important, and for this purpose, the use of a facile and cost‐effective method for synthesizing ultrabright SiQDs, such as the hydrogen silsesquioxane (HSQ) polymer method, is crucial.^[^ ^16^ ^]^

Solvent engineering is a simple and powerful method that has been used to enhance the performance of various QD LEDs, and the choice of solvent as QD dispersant during LED fabrication has been shown to significantly affect LED performance. For instance, for CdSe/ZnS QD LEDs, changing the solvent used for preparation to one that was orthogonal to the underlayer materials yielded two‐fold increases in the EQE and EL intensity.^[^ ^52^ ^]^ Using ternary solvents for CsPbX_3_ (X: halogen) perovskite LED fabrication reduced the coffee‐ring effect, resulting in four‐fold increases in the EQE and EL intensity.^[^ ^53^ ^]^ A significant increase in the operating half‐life, from 33 to 25 178 h, was realized for CdZnSe/ZnS QD LEDs by using a 1‐cyclohexyl‐ethanol/octane/*n*‐butyl acetate solvent mixture for the QD layer preparation step of LED fabrication.^[^ ^54^ ^]^ Solvents capable of dissolving hole‐transport layer (HTL)^[^ ^55^ ^]^ and/or electron‐transport layer (ETL) materials^[^ ^56^ ^]^ have also been shown to produce improved film morphologies, resulting in better QD LED performance. Therefore, despite being a very simple strategy, solvent engineering allows significantly enhanced performance and lifetimes to be realized.

However, thus far, solvent engineering has not been actively applied to SiQD LED fabrication. Toluene is the most popular dispersant, and almost all colloidal SiQDs, which have surfaces passivated by hydrocarbon molecules, are stable for long periods (≈1 year) when stored as toluene dispersions. Hence, many SiQD synthesis and LED fabrication researchers have adopted this solvent as their preferred dispersant. In contrast, solution‐processed QD LED production is affected by not only QD dispersity but also by substrate compatibility and wettability, as well as dispersant vapor pressure. In fact, one of the highest SiQD LED EQEs (8%) was obtained by using SiQDs dispersed in chloroform,^[^ ^31^ ^]^ possibly because chloroform partially dissolved the HTL, improving the quality of the heterojunction between the HTL and SiQDs. In addition, hydrocarbon solvents such as octane have been commonly used in Cd‐ and In‐based QD LEDs and perovskite QD LEDs, yielding high‐performance QD LEDs.^[^ ^57^, ^58^, ^59^ ^]^ However, as toluene is a good dispersant, minimizing SiQD aggregation, and because it has higher dispersity than other hydrocarbon solvents for long‐term storage, the use of other dispersants has not been actively considered. Therefore, solvent engineering of SiQD LEDs is an unexplored area. However, if high‐performance SiQD LEDs with long‐term stability were to be achieved via solvent engineering, the realization of this simple strategy would be a milestone in the development of next‐generation efficient, stable, and sustainable QD LEDs.

In this article, we report SiQD LEDs with a maximum EQE of 16.5% and an EL center wavelength in the far‐red (*λ*_EL_ = 750 nm). This is a new record for SiQD LED EQE, and SiQD LED lifetimes as much as 733 times greater than the previous record, and we also achieved record‐breaking luminance at a voltage five times lower than that required for the previous luminance record, all thanks to solvent engineering. A total of 27 SiQD LEDs were fabricated using four different solvents: toluene, chloroform, octane, and decane. Stochastic data analyses, including current–voltage–light (*I*–*V*–*L*) curve, EQE, film morphology, PL mapping, contact angle, and EL stability analyses, indicated that the choice of solvent for preparing the emissive layer of the LED was critical. SiQD LEDs with luminances comparable to or higher than those of state‐of‐the‐art perovskite QD LEDs were developed. Our investigations also revealed the key processes and mechanisms underlying the effectiveness of this solvent engineering approach for developing efficient and long‐term‐stable SiQD LEDs.

## 2 Results and Discussion

### 2.1 Structure and Optical Properties of Dodecyl‐Terminated SiQDs

Colloidal SiQDs were synthesized using the facile and cost‐effective HSQ polymer method,^[^ ^16^, ^60^ ^]^ followed by surface passivation using 1‐dodecene via thermal hydrosilylation. The synthesis procedures are described in Section S1, Supporting Information.

**Figure 1:**
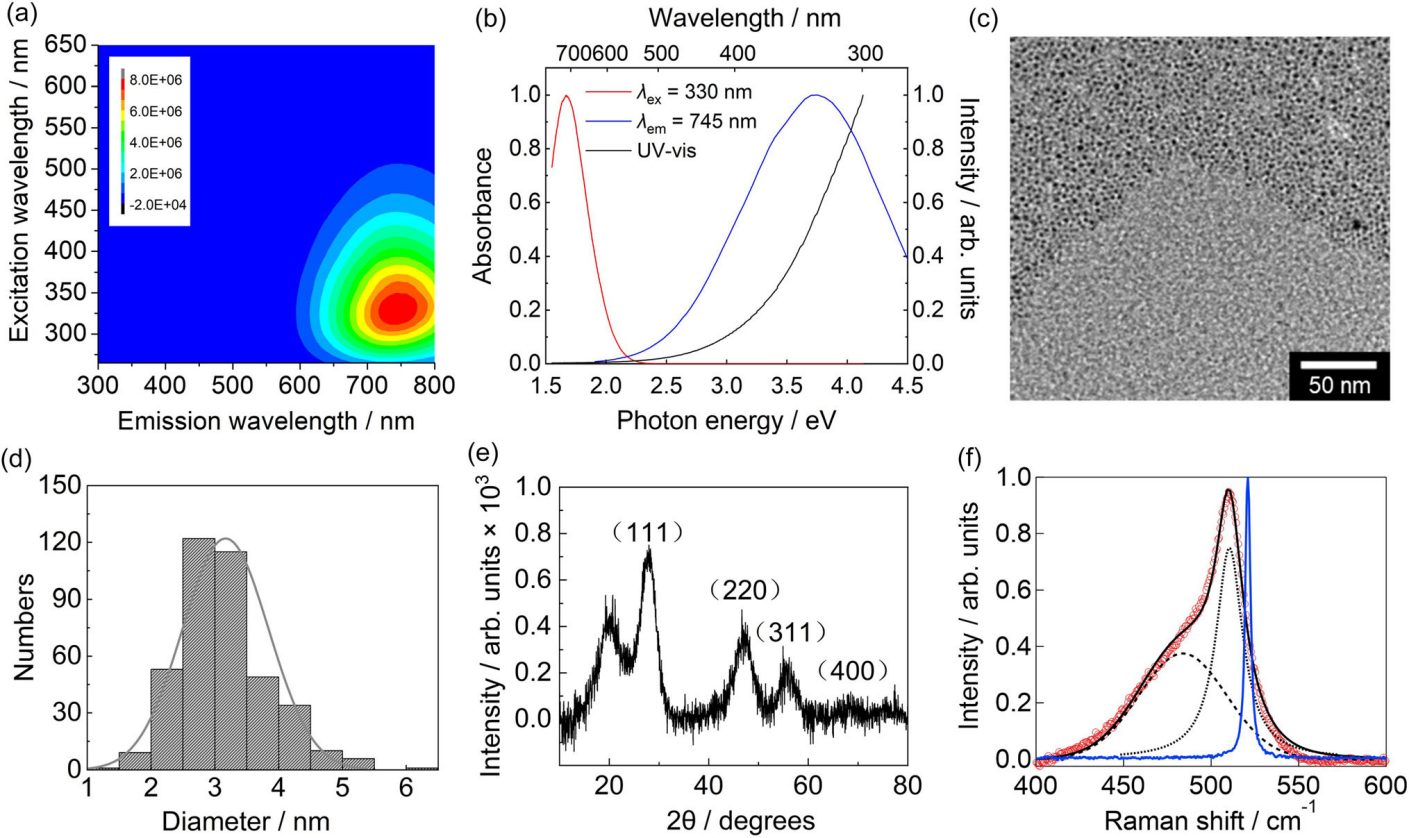
Properties and structures of dodecyl‐terminated SiQDs dispersed in chloroform. a) 2D PL spectrum; the horizontal axis, vertical axis, and color scale indicate the emission wavelength, excitation wavelength, and intensity, respectively. b) PL (red line, measured with excitation at 330 nm), PLE (blue line, measured at an emission wavelength of 745 nm), and UV–vis absorption (black line) spectra. c) TEM image. d) Size distribution obtained via analysis of the particles in the image in (c). The average size is 3.2 ± 0.7 nm (mean ± SD, *n* = 400). e) XRD pattern. The indices overlaid on the pattern are the Miller indices of crystalline Si planes assigned to the various peaks. The peak at 22° is attributed to SiO_2_. f) Raman spectrum (red open circles) and fit result (black solid line); the contributions to the fit of the crystalline transverse optical (TO) mode band (dotted black line) and amorphous band (dashed black line), which were modeled using Lorentzian and Gaussian functions, respectively, are also shown. The blue line is the Raman spectrum of a Si wafer.

**Figure** **1**a shows the 2D PL spectrum of a chloroform dispersion of the prepared dodecyl‐terminated SiQDs. The highest PL intensity (at a wavelength of 745 nm in the far‐red) occurs when the excitation wavelength is 330 nm; the corresponding PLQY was obtained as 63%, and the PLQYs of the other dodecyl‐terminated SiQDs—those dispersed in decane, octane, or toluene—were similar (Table S1, Supporting Information). The PLQY value of 63% that we obtained is high in comparison with values in the literature: typical previously reported PLQY values are in the range of 10–40%,^[^ ^15^ ^]^ with another high value (PLQY ≈ 80%) having been obtained previously using the HSQ polymer method.^[^ ^16^ ^]^ It should be emphasized that our high PLQY was achieved for SiQDs with a high crystallinity (90%), which were obtained from precursor HSQ polymers with relative cage component contents of greater than 65% and relative Si—H functional group contents greater than 40%.^[^ ^16^ ^]^

Figure 1b shows PL and PL excitation (PLE) spectra, which correspond to specific cut‐throughs of the 2D spectrum in Figure 1a, as well as the UV–vis absorption spectrum. The PL (red line) and PLE (blue line) spectra displayed were acquired with excitation at 330 nm and by monitoring the emission at 745 nm, respectively; these spectra are plotted using a linear wavelength axis in Figure S1a, Supporting Information. Of particular note is the fact that there is little absorption (a small absorption tail) in the wavelength range of 650–750 nm (Figure 1b; Figure S1a, Supporting Information). In addition, the PLE spectrum also features low intensities in the same wavelength range. Hence, excitation at 650–750 nm cannot provide sufficient PL intensity. This is because silicon has a strong absorption in the UV (≈350 nm) owing to the direct Γ–Γ transition, whereas PL is observed via the indirect (X–Γ) transition.^[^ ^61^ ^]^ Thus, a significant Stokes shift occurs, and both the absorption and the PLE intensities significantly decrease in the wavelength range of 650–750 nm. This feature is commonly observed in the optical spectra of SiQDs.^[^ ^9^, ^10^, ^11^, ^15^, ^16^, ^18^, ^19^, ^21^ ^]^ As another crucial result, it was verified that the solvent effect on the PL spectral profiles was negligible (Figure S2, Supporting Information).

Figure 1c,d shows a transmission electron microscopy (TEM) image of the dodecyl‐terminated SiQDs and the corresponding distribution of SiQD sizes, and an expanded TEM image is displayed in Figure S1b (Supporting Information); the average SiQD size was found to be 3.2 ± 0.7 nm (mean ± SD, *n* = 400; standard deviation [SD]). Figure 1e shows the X‐ray diffraction (XRD) pattern, the form of which indicates that the SiQDs were crystalline. This was further verified by means of a Raman spectroscopic analysis (Figure 1f), and the crystallinity was quantified as 84% based on spectral fitting and the scattering cross sections (Section S2, Supporting Information). The Fourier‐transform infrared spectroscopy spectrum (Figure S3, Supporting Information) verifies the presence of Si–dodecyl, Si—H, and Si—O—Si functional groups in the SiQDs, and it includes features typically observed in the spectra of SiQDs synthesized using the same method.^[^ ^39^, ^41^ ^]^ The respective coverages of these groups were quantified as 18.3 ± 1.2%, 14.0 ± 4.5%, and 67.7 ± 3.9% (mean ± SD, *n* = 3 in each case), based on an analysis of the integrated intensities and absorption cross sections of the bands (Section S2, Supporting Information).

### 2.2 Performance of SiQD LEDs

**Figure 2:**
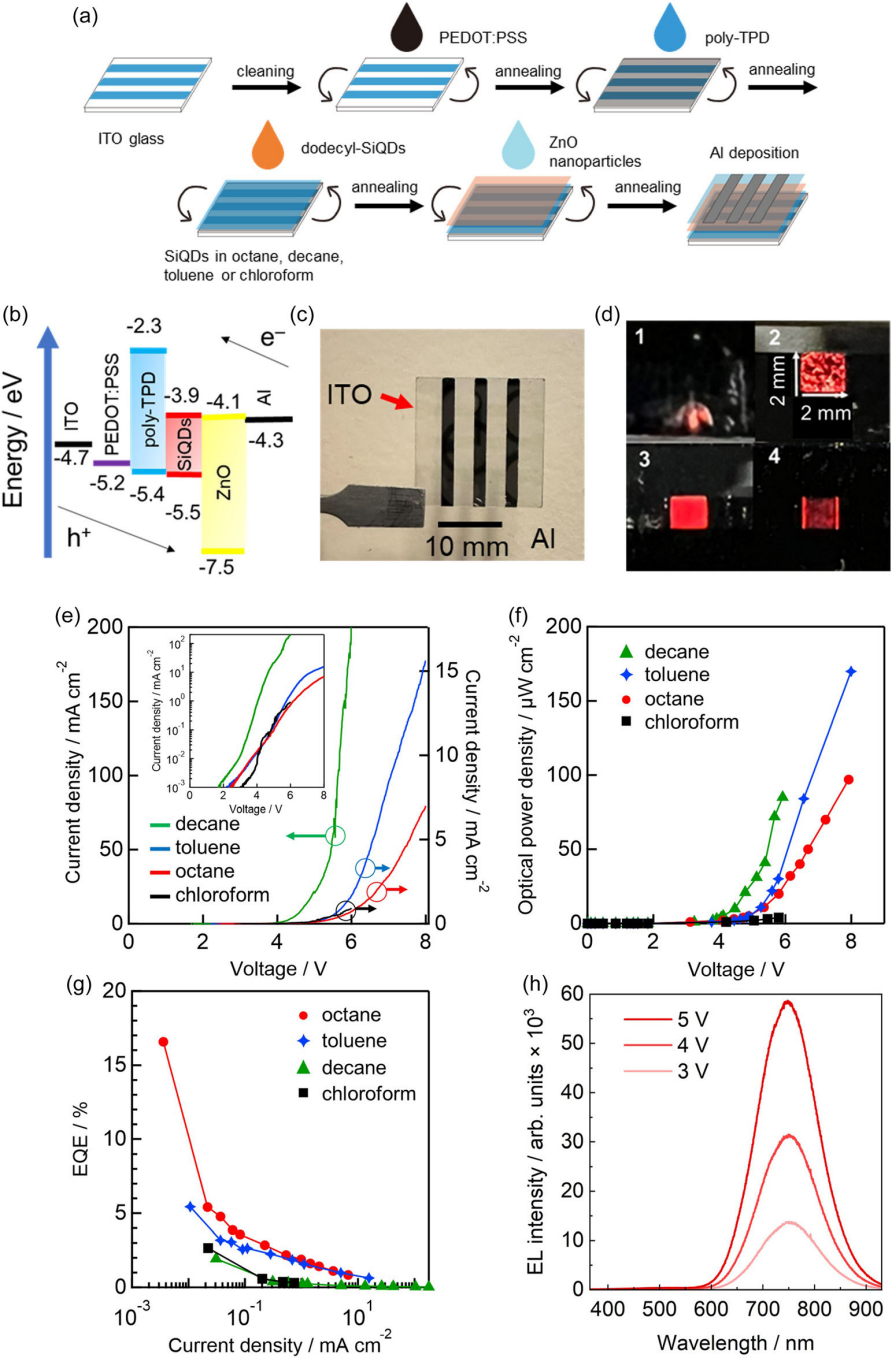
SiQD LEDs fabricated using dodecyl‐terminated SiQDs via solution processing. a) Schematic illustration of fabrication procedures. b) SiQD LED energy diagram showing the energy bands of each of the layered materials. c) Photograph of SiQD LED with nine active areas. The arrows indicate the positions of the ITO anode (three 2 mm wide horizontal lines) and Al cathode (three 2 mm wide vertical lines). d) Photographs showing the EL of SiQD LEDs fabricated using 1) chloroform, 2) decane, 3) octane, and 4) toluene as the SiQD dispersants (chloroform‐, decane‐, octane‐, and toluene‐dispersed SiQD LEDs). The size of the active area was 2 × 2 mm in each case. e) Current density *I* versus voltage *V*; the insets show the same data plotted using a log scale for the vertical axis. f) Optical power density *L* versus applied voltage *V*. g) EQE versus current density *I*. h) EL spectra of octane‐dispersed SiQD LED measured at 3, 4, and 5 V. Note that the EL spectrum measured at 3 V corresponds to the maximum EQE (16.5%) data point in (g).

**Figure** **2**a shows the fabrication procedure for the SiQD LEDs and Figure 2b shows the energy diagram for the multilayer materials (see Section S3, Supporting Information, for further details of the fabrication methods). Briefly, dodecyl‐terminated SiQDs dispersed in each solvent (toluene, chloroform, octane, or decane) were spin‐coated onto an HTL made from poly [*N*,*N*′‐bis(4‐butylphenyl)‐*N*,*N*′‐bis(phenyl)‐benzidine] (poly‐TPD) to prepare each SiQD LED. The remaining steps in the LED fabrication procedure (all steps other than the abovementioned colloidal SiQD spin‐coating process) were the same for all four types of SiQD LED. A total of 27 SiQD LEDs were prepared in the present study: 5, 10, 6, and 6 SiQD LEDs were prepared using the chloroform, toluene, octane, and decane dispersions, respectively. Herein, we refer to each of these different types of SiQD LED as the tolune‐dispersed, chloroform‐dispersed, octane‐dispersed, and decane‐dispersed SiQD LEDs, respectively.

Figure 2c shows a photograph of one of the SiQD LEDs prior to operation and Figure 2d shows photographs of examples of the four different types of SiQD LED during operation (active area: 2 × 2 mm), under an applied voltage; the EL of each LED is clearly visible. Significantly different EL features can be observed in the photographs, and the most homogeneous spatial EL distribution is apparent for the octane‐dispersed SiQD LED.

In Figure 2e–h, we present the optoelectronic characteristics of four SiQD LEDs fabricated using dodecyl‐terminated SiQDs dispersed in different solvents. The reproducibility of the data was verified by performing three repeat measurements in each case, and the data from the repeat measurements are shown in Figure S4 (Supporting Information). The performance of the four SiQD LEDs was strongly dependent on the solvent used as the dispersant for the dodecyl‐terminated SiQDs. The current density versus applied voltage (Figure 2e), optical power density versus applied voltage (Figure 2f), and EQE versus current density (Figure 2g) profiles varied significantly depending on the solvent. In other words, the process of spin‐coating the SiQD solution onto the poly‐TPD HTL was critical in determining the performance of the SiQD LEDs.

Figure 2h shows the EL spectra of an octane‐dispersed SiQD LED. This EL spectrum, with a peak wavelength of 750 nm in the far‐red, is similar to the PL spectrum, which peaks at 745 nm (Figure 1b; Figure S1a, Supporting Information). The EL intensity increased with the applied voltage, but the spectral profile was otherwise unchanged. Sidebands were not observed in any of the EL spectra, indicating that no materials other than the SiQDs contributed to the observed EL spectra. Therefore, the fully intact SiQD layer without pinholes, sandwiched between the upper and lower layers within the assembled SiQD LEDs, provided a confined SiQD sheet in which electron–hole recombination was facilitated. In addition, the EL spectra of the decane‐ and toluene‐dispersed SiQDs were similar to that of the octane‐dispersed SiQD LED, but the chloroform‐dispersed SiQD LED exhibited a low EL intensity (Figure S5, Supporting Information). These results follow the same trend as the SiQD LED performance results (Figure 2e–g): the chloroform‐dispersed SiQD LED was characterized by low current density, optical powder density, and EQE.

In Figure 2e, the current varies across four orders of magnitude among the four SiQD LEDs. The optical power density (EL intensity) plot (Figure 2f) indicates that the decane‐dispersed SiQD LED required the highest current density to emit EL with a high optical power density, whereas the chloroform‐dispersed SiQD LED showed the lowest current density, resulting in the lowest optical power density (negligible EL intensity). In contrast, the octane‐ and toluene‐dispersed SiQD LEDs exhibited similar optical power densities, but the former exhibited a lower current density (more efficient EL). These differences are noteworthy at low current density but are dramatically diminished in the 1–10 mA cm^−2^ range (Figure 2g; Figure S4, Supporting Information).

For a more quantitative analysis, we evaluated the EQE based on a previously reported method,^[^ ^16^, ^39^, ^41^ ^]^ as detailed in Section S4, Supporting Information. The obtained SiQD LED EQEs are plotted as a function of the current density in Figure 2g. The highest EQE was obtained as 16.5% for the octane‐dispersed SiQD LEDs. Note that this value is the highest EQE of any SiQD LED reported in the literature, to the best of our knowledge;^[^ ^16^, ^27^, ^28^, ^29^, ^30^, ^31^, ^32^, ^33^, ^34^, ^35^, ^36^, ^37^, ^38^, ^39^, ^40^, ^41^, ^42^, ^43^, ^44^, ^45^, ^46^ ^]^ in Table S2, Supporting Information, we present a comprehensive list of all the available literature SiQD LED EQE data for comparison. In addition, the average EQE for our optimized SiQD LEDs was 11%, based on a stochastic analysis, and this excellent performance was realized by using octane as the dispersant for the SiQDs, as detailed in Section 2.4, 2.5. Furthermore, note that a good EL spectral profile was obtained at EQE = 16.5%. Specifically, the measured EL spectrum corresponding to the first point in Figure 2g had a profile the same as those measured at higher current densities (Figure 2h).

### 2.3 Record Luminance in the Far‐Red

In addition, it should be noted that compared with all the results in the literature, higher luminance, 66 cd m^−2^, was obtained for the SiQD LEDs we fabricated in the present study even though we used a voltage (6 V) five times lower than the previous luminance record (58 cd m^−2^ at 30 V).^[^ ^32^ ^]^ In Table S2 and S3, Supporting Information, a full comparison of the literature results is given, and in Figure S6, Supporting Information, the luminance profile as a function of current density is displayed.

Furthermore, it should be mentioned that the luminance values obtained in the present study were for EL at redder wavelengths (*λ*_EL_ = 750 nm, in the far‐red) with respect to the EL wavelength observed in the aforementioned previous study (*λ*_EL_ = 685 nm, in the red). The candela quantifies the light intensity in the 380–780 nm wavelength region, taking into account human vision, based on the chromaticity (CIE) diagram.^[^ ^62^, ^63^ ^]^ Therefore, at the same optical power density (W m^−2^), a typical luminance measurement in cd m^−2^ is lower in the far‐red region than, for example, one in the green light region by a factor of several hundreds to tens of thousands (see Figure S8, Supporting Information). Hence, the portion of the EL intensity of our SiQD LEDs in the 780–930 nm wavelength range did not make a significant contribution to the luminance. Indeed, although high luminance values, in the range of 100–10 000 cd m^−2^ in the blue, green, and red (650–700 nm) EL spectral regions, have been reported for In‐based, Cd‐based, and perovskite QD LEDs,^[^ ^64^, ^65^, ^66^ ^]^ the luminance values of state‐of‐the‐art perovskite QD LEDs in the far‐red wavelength region (700–800 nm) are much lower; for example, values of 180 cd m^−2^ (*λ*_EL_ = 735 nm)^[^ ^67^ ^]^ and <4.3 cd m^−2^ (*λ*_EL_ = 772 nm)^[^ ^68^ ^]^ have been reported (Section S5, Supporting Information). In addition, to the best of our knowledge, LEDs with high luminance in the far‐red have not been achieved using Cd‐based or In‐based QDs because the bandgap energies of these materials in the bulk (e.g., CdSe and InP, 1.4–1.8 eV) are significantly greater than that of silicon (1.1 eV).

In conclusion, the SiQD LEDs fabricated in this study exhibited very high luminance in the far‐red (66 cd m^−2^, *λ*_EL_ = 750 nm), comparable to state‐of‐the‐art perovskite QD LED luminance. This record luminance achieved in the far‐red region marks a significant step forward in SiQD LED research. In fact, these high‐luminance far‐red SiQD LEDs can find applications as nontoxic light sources for use in vegetable and/or fruit growing in greenhouses because far‐red light is known to significantly increase growth rates.^[^ ^69^, ^70^ ^]^ In addition, stable high‐luminance far‐red QD LEDs are increasingly being seen as invaluable light sources for photodynamic therapy, to kill tumors via singlet oxygen generation.^[^ ^71^, ^72^, ^73^ ^]^ Moreover, it should be noted that there is significant potential for further increases in SiQD LED luminance because this is the first time that solvent engineering has been applied to the design and fabrication of these devices.

### 2.4 Dependence of SiQD LED Performance on Solvent Used as Dispersant

As all the SiQD LEDs were fabricated using the same procedures except for the choice of dispersant for the SiQD emissive layer, to study the mechanism by which we were able to achieve record‐breaking efficiency, luminance, and voltage, we focused on the SiQD layers of the SiQD LEDs. Accordingly, SiQD films were prepared by spin‐coating each of the four SiQD dispersions on poly‐TPD films (HTLs), and the resultant surfaces were characterized using optical microscopy and PL spectroscopic mapping.

**Figure 3:**
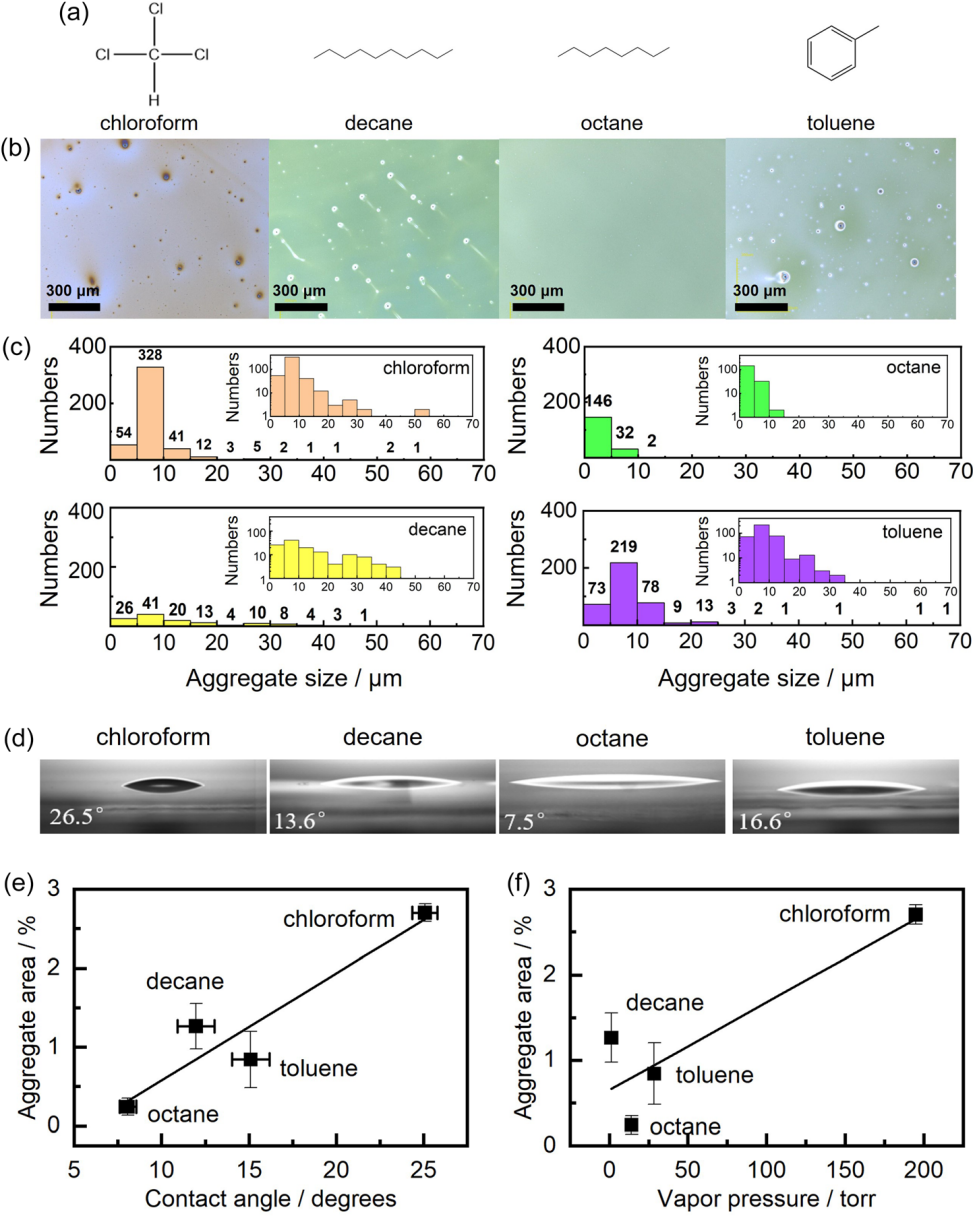
Solvent dependence of SiQD film morphology. a) Molecular structures of four solvents used as dispersants for the dodecyl‐terminated SiQDs. b) Optical micrographs of SiQD layers prepared by spin‐coating the poly‐TPD layers of LEDs with the different SiQD dispersions. c) SiQD aggregate size distributions determined by analyzing the images shown in (b); the insets show the same data plotted using a log scale for the vertical axis. d) Photographs of drops of each of the four SiQD dispersions on the poly‐TPD layer of the SiQD LED; a typical contact angle measurement result is overlaid on each image. Aggregate area versus, e) contact angle of the dispersion at 23 °C, and f) vapor pressure of the dispersant at 25 °C. To verify the repeatability of these results, aggregate area measurements and analyses were conducted three times, and the means and standard deviations are presented as the data points and error bars, respectively, in (e) and (f). Error bars in (e) and (f) denote mean ± SD (*n* = 3 for each sample).

**Figure** **3**a shows the molecular structures of the solvents used as dispersants and Figure 3b shows micrographs of the corresponding SiQD films. Inhomogeneous morphologies, including island‐like structures, were observed for all the films, but the size and number of the islands varied significantly between the samples. To verify the composition of these island structures on the HTL, we performed PL mapping measurements, using a protocol described elsewhere for previous studies.^[^ ^74^, ^75^, ^76^, ^77^, ^78^, ^79^, ^80^ ^]^ The PL spectra acquired from points within these morphological features were found to be almost identical to that of the dodecyl‐terminated SiQDs, and hence it was confirmed that the islands consisted of SiQD aggregates (Figure S9 and S10, Supporting Information).

To further investigate the mechanism behind the multiple‐record‐breaking performance of the SiQD LEDs, the numbers and sizes of the SiQD aggregates formed in the four SiQD films on the HTL were quantified via image analysis (Figure 3c). The obtained histograms indicate that the SiQD film prepared by spin‐coating using the chloroform dispersion of SiQDs produced the greatest number of SiQD aggregates. In contrast, the SiQD film prepared by spin‐coating using a decane dispersion of SiQDs was characterized by a small number of SiQD aggregates that included several large ones (20–50 μm). Note that the SiQD film prepared by spin‐coating using octane as the dispersant featured a small number of SiQD aggregates, and there were no large (>15 μm) aggregates (an expanded view of the image is shown in Figure S11, Supporting Information). These observations and analyses were carried out three times, and additional data are shown in Figure S12 and S13 (Supporting Information). It should be noted that the SiQD dispersions used for spin‐coating contained no SiQD aggregates because just before LED fabrication, each SiQD dispersion was filtered using a cartridge filter with a pore size of 0.45 μm; therefore, we concluded that SiQD aggregation occurred during SiQD layer formation.

To further explore the differences in SiQD aggregate formation, we measured the contact angles of the four SiQD dispersions on the HTL (Figure 3d). Contact angles *θ* of 25.1° ± 1.2° (chloroform dispersion), 15.1° ± 1.9° (toluene dispersion), 12.0° ± 1.8° (decane dispersion), and 8.1° ± 0.8° (octane dispersion) were obtained based on three repeat measurements (mean ± SD, *n* = 3 for each sample). The SiQD aggregate area was plotted versus the measured contact angle (Figure 3e) and solvent vapor pressure (Figure 3f); the SiQD aggregate area was obtained as the sum of the areas of all the SiQD aggregates in the measured area (1.34 × 1.34 mm) divided by the same area (Section S4, Supporting Information). The octane dispersion of SiQDs, characterized by the smallest contact angle, produced a film with homogeneous morphology and the smallest SiQD aggregate area. Therefore, the SiQD aggregate area was proportional to the magnitude of the contact angle and vapor pressure, which significantly affected the performances of the four types of SiQD LED. Namely, the use of a solvent with a small contact angle and low vapor pressure as dispersant hindered SiQD aggregate formation, yielding smooth SiQD films that, when incorporated into the LED structure, realized enhanced SiQD LED performance.

**Figure 4:**
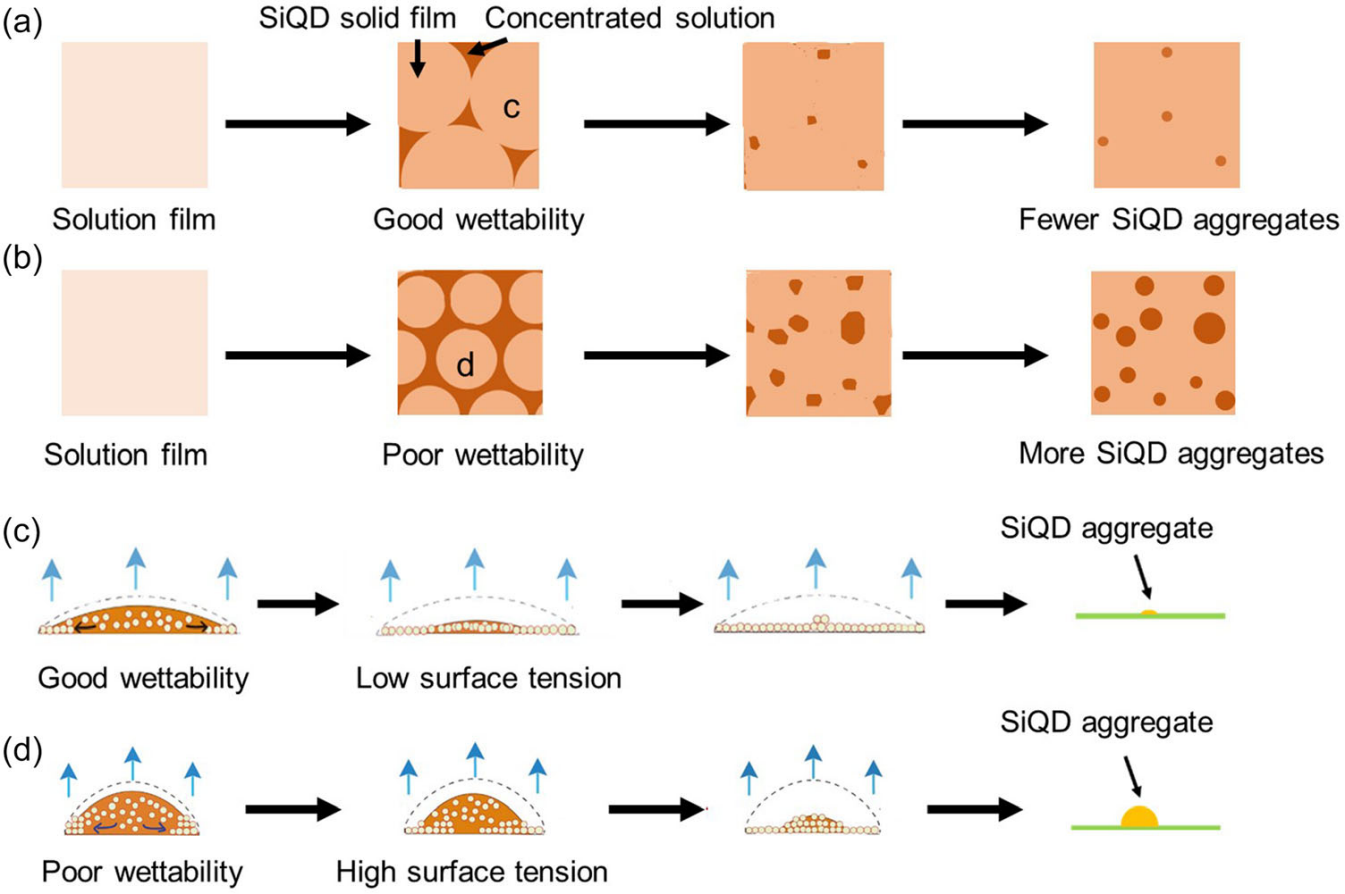
SiQD aggregate formation during the drying of the SiQD dispersion on the poly‐TPD film and bubble formation during SiQD LED operation. Schematic top views of films with a) large and b) small SiQD droplets, produced by drying dispersions with small and large contact angles, respectively. Schematic side views of c) large and d) small droplets.

We now present a mechanism to explain how the dispersant affected the SiQD aggregation behavior and hence also the performance of the SiQD LEDs. Immediately after spin‐coating, a thin SiQD dispersion film was formed on the HTL (left‐most images in **Figure** **4**a,b). When the contact angle was small, wetting of the HTL by the SiQD dispersion occurred, and a large region of solid SiQD film was formed during the drying process (Figure 4a). This large region was eventually converted into a large homogeneous SiQD film on the HTL (Figure 4c), although at the outer perimeter of the region, during drying, the SiQD solution was concentrated via the so‐called coffee‐ring effect, resulting in SiQD aggregation. Conversely, when a SiQD dispersion with a large contact angle (the dispersions prepared using one of the other three solvents) was used, small, thick areas of film were generated, and large SiQD aggregates were formed inside these areas (Figure 4d; Figure S12 and S13b,c, Supporting Information). Therefore, such SiQD dispersions favor aggregate formation inside and at the edges of film areas, and the SiQD LEDs fabricated from these films are characterized by poor efficiency and stability.

Finally, we mentioned another important chemical effect of the SiQD dispersants on film (HTL) formation and hence also on the performance of the resulting LEDs. Among the various LEDs, the color of the chloroform‐dispersed SiQD LED is different (Figure 3b; Figure S13, Supporting Information). This could be because of a difference in HTL thickness. To examine this possibility, we measured UV–vis absorption spectra of each HTL (poly‐TPD) on the poly(3,4‐ethylenedioxythiophene) polystyrene sulfonate (PEDOT:PSS) film before and after spin‐coating using the four dispersants, concluding the HTL thickness after spin‐coating using chloroform was significantly lower (Figure S14a, Supporting Information). Therefore, we next prepared four SiQD LEDs with different HTL thicknesses such that, after some dissolution of the HTL during spin‐coating with the SiQD dispersions, the HTL thicknesses were the same (Figure S14b, Supporting Information). We measured the *I*–*V* curves and evaluated the performances of these four SiQD LEDs with the same HTL thickness (Figure S15, Supporting Information). Good diode profiles were obtained for the octane‐ and decane‐dispersed SiQD LEDs, but poor ones were obtained for the chloroform‐dispersed and toluene‐dispersed SiQD LEDs. Thus, the former two SiQD LEDs exhibited better performance at the same poly‐TPD film thickness. In conclusion, it was verified, considering the chemical effect of HTL dissolution as well as the homogeneity of the SiQD layer formed with few aggregates, that octane was the best dispersant for preparing the present SiQD LEDs.

### 2.5 Mechanism Underlying Excellent Performance of SiQD LEDs Clarified by Stochastic Analysis

**Figure 5:**
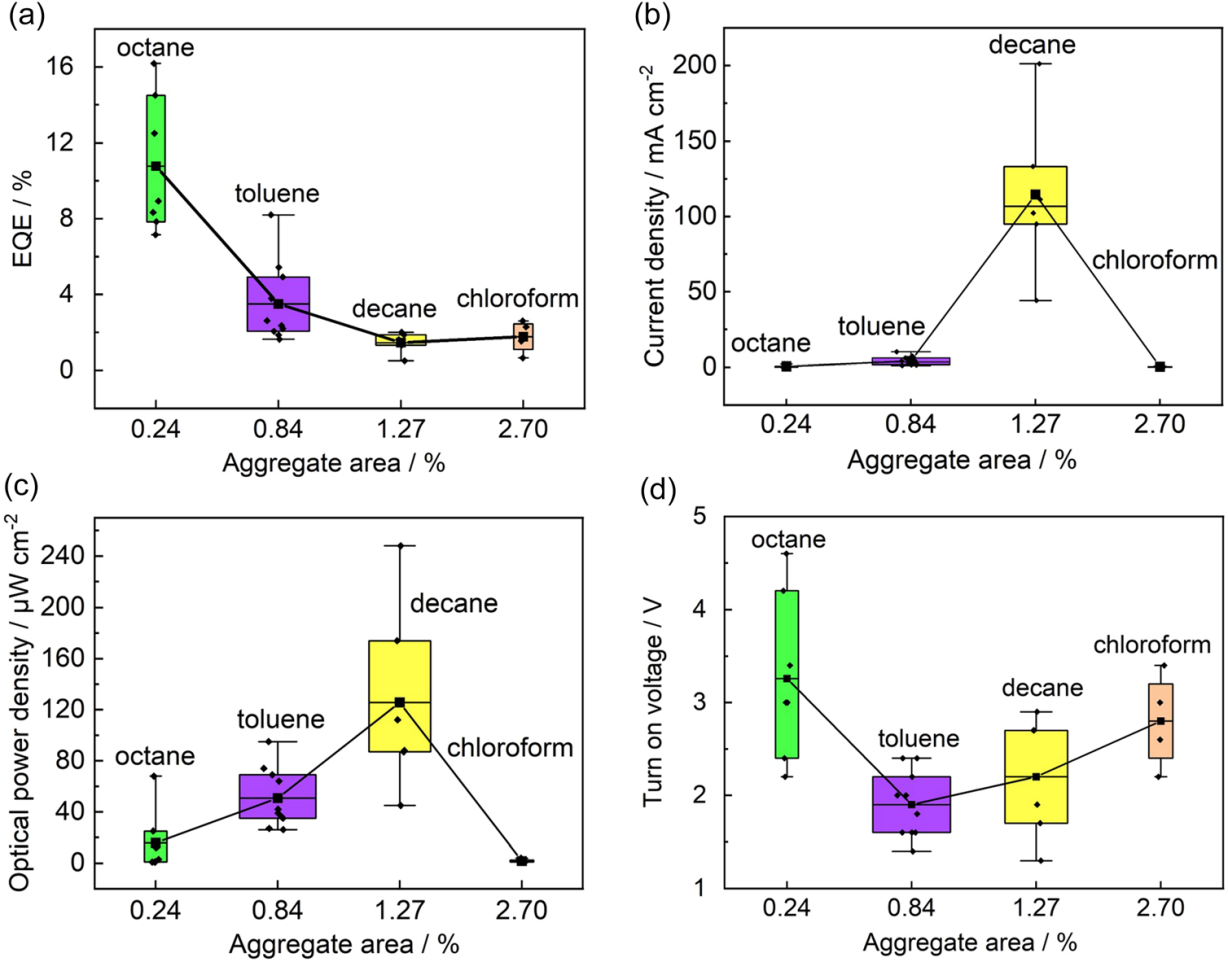
Box‐and‐whisker plots illustrating the optoelectronic performances of the four types of SiQD LED. These data were obtained by analyzing the performance and characteristics of 27 SiQD LEDs. Error bars in (a–d) denote mean ± SD; *n* = 6 (octane‐dispersed SiQD LEDs), *n* = 10 (toluene‐dispersed LEDs), *n* = 6 (decane‐dispersed LEDs), and *n* = 5 (chloroform‐dispersed LEDs). The width of each box represents the standard deviation of the aggregate area (*n* = 3 for each sample in Figure 3). a) Maximum EQE versus aggregate area. Note that the EQE values plotted are maximum values obtained by varying the current density. b) Current density measured at an applied voltage of 6 V versus aggregate area. c) Optical power density measured at 6 V versus aggregate area. d) Turn‐on voltage (voltage at 0.001 mA cm^−2^) of each device versus aggregate area. The squares indicate the mean values for each type of LED.

To better understand the performances of the different devices, considering repeatability and reproducibility, we performed a stochastic analysis of 27 SiQD LEDs, creating box‐and‐whisker plots of the EQE, current density, optical power density, and turn‐on voltage versus the total SiQD aggregate area on the HTL for the different SiQD LEDs (**Figure** **5**a–d). It is apparent from the figure that the highest individual EQE value was obtained for one of the octane‐dispersed SiQD LEDs (16.5%), and the highest average EQE was also obtained for this type of LED (11%), as well as the lowest average aggregate area and smallest range of aggregate sizes. In contrast, the EQEs, current densities, and optical power densities of the chloroform‐dispersed SiQD LEDs were the lowest, and the aggregate area in the SiQD layer in these devices was highest. Thus, the formation of a low‐quality film with many SiQD aggregates resulted in poor SiQD LED performance. In addition, chloroform was a good solvent for the poly‐TPD (HTL), and hence it dissolved the part of HTL during the SiQD film formation process, which had a deleterious effect on the performance of the LED (Figure S14 and S15, Supporting Information), as described in Section 2.4. Therefore, the choice of solvent used to disperse the SiQDs was critical for obtaining smooth and homogeneous SiQD and HTL films and high‐performance SiQD LEDs. In conclusion, among the solvents we studied, octane was found to be the best dispersant, producing the most efficient SiQD LEDs, and the use of chloroform resulted in the most inefficient SiQD LEDs owing to the formation of large numbers of SiQD aggregates on the HTL and the HTL being damaged.

However, it was observed that the highest current densities (Figure 5b), and hence also the highest optical power densities (Figure 5c), were measured for the decane‐dispersed SiQD LEDs, along with a low turn‐on voltage, but these devices were characterized by low EQEs and featured relatively large SiQD aggregate areas on their HTLs. Conversely, the octane‐dispersed SiQD LEDs had the highest turn‐on voltage (Figure 5d), indicating a high voltage was required for carrier migration, but the EQEs of these devices were the highest and their SiQD aggregation was negligible. Taking together all the results shown in Figure 5, in the decane‐dispersed SiQD LED, SiQD aggregation produced bright EL with a high current density and low turn‐on voltage; this was associated with a low EQE owing to the inefficient conversion of injected carriers to photons. In contrast, for the devices featuring minimal SiQD aggregation (the octane‐dispersed SiQD LEDs), the current density was lower and the turn‐on voltage greater, but these SiQD LEDs had the greatest EL conversion efficiencies, i.e., the highest EQE. In conclusion, the quality of the SiQD film deposited on the poly‐TPD film (HTL) determined the performance of the SiQD LED.

Next, we discuss in detail the origins of the SiQD LED performance by considering the multilayer structures and film morphologies, based on all the results shown in Figure 3, 4, 5. Efficient carrier migration in the conjugated‐polymer poly‐TPD with high electronic conductivity was hindered by the electronic insulator ligands of the SiQDs, which entirely covered the HTL when a homogeneous SiQD film (large‐area heterojunction) was formed (Si–dodecyl and Si—O—Si ligand coverage on the SiQD surface: 85%; Section 2.1). Hence, for the octane‐dispersed SiQD LEDs, a high EL conversion efficiency and high turn‐on voltage were observed (Figure 5a,d). In contrast, in the case of the decane‐dispersed SiQD LEDs, heterogeneous SiQD deposition with a high degree of aggregation (small‐area heterojunction formation) reduced the conversion efficiency (Figure 5a) but promoted carrier migration in the conjugated‐polymer poly‐TPD layer, owing to the fact that there were fewer regions of SiQDs with electronic insulator ligands, resulting in a high current density (and a high optical power density) with a low turn‐on voltage (Figure 5b–d).

Moreover, the large SiQD aggregates in the decane‐dispersed SiQD film produced pinholes in the ZnO ETL (Figure S16, Supporting Information), which promoted carrier migration through the ZnO energy barrier, resulting in increased current density. This conclusion was further verified based on an analysis of the SiQD aggregate volume distributions (Figure S12 and S13c, Supporting Information). Significant numbers of 20–50 μm SiQD aggregates were observed on the HTL of the decane‐dispersed SiQD LED, and aggregates in this size range may be responsible for the observed high current density owing to the generation of pinholes in the ZnO ETL. These large SiQD aggregates were also responsible for the highest leakage current for decane‐dispersed SiQD LED (Figure S17, Supporting Information). In addition, fewer and even fewer amounts of SiQD aggregates with the aforementioned sizes were observed on the HTLs of the toluene‐ and octane‐dispersed SiQD LEDs, respectively, and low current densities (Figure 5b) owing to small amounts of pinholes, resulting in higher EQEs, were obtained for these LEDs (Figure 5a). In fact, it was observed that the octane‐dispersed SiQD LED, with its homogeneous SiQD film without SiQD aggregates, featured the lowest leakage ratio (Figure S17, Supporting Information). In conclusion, the SiQD dispersant was critically important in determining the morphology of the emissive SiQD layer and the damage to the HTL and ETL, and octane was demonstrated to be the best dispersant for preparing far‐red SiQD LEDs.

### 2.6 SiQD LED Stability and Degradation Mechanism

**Figure 6:**
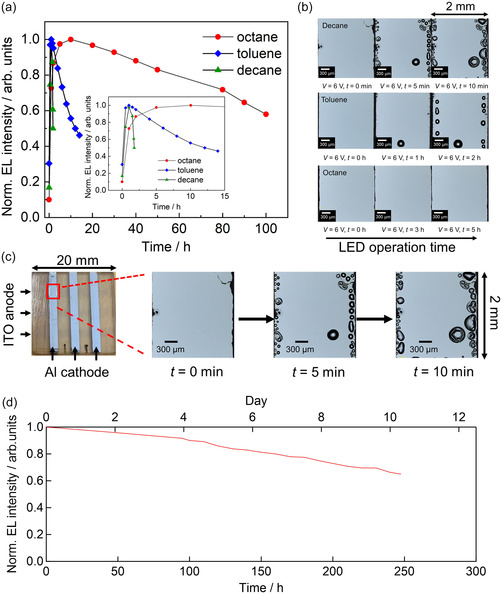
Operation lifetimes of three SiQD LEDs. a) EL intensity as a function of device working time. The data for the decane‐, octane‐, and toluene‐dispersed SiQD LEDs were measured at applied voltages of 3, 6, and 6 V, respectively. The decane‐dispersed SiQD LED, which has a high current density, was degraded very quickly, preventing accurate lifetime measurements at 6 V, and hence measurement of its lifetime was conducted at 3 V. In each case, the conditions for the lifetime measurements were as follows: 23 °C temperature, 50% relative humidity, air atmosphere, and the EL intensity was measured in a dark box. b) Optical micrographs of LEDs before and after the application of 6 V for different periods of time. These images were captured using ten‐fold magnification. c) Optical micrographs showing the process of bubble formation overtime at the edge of the Al cathode in the active area of a SiQD LED. The three rightmost photographs in (c) show the decane‐dispersed SiQD LED and are the same as those shown in Figure 6b. d) EL intensity as a function of device working time for the improved octane‐dispersed SiQD LED measured at applied voltage of 6 V; the improvement was realized by changing the annealing conditions for the preparation of the ZnO ETL film.

Finally, we discuss the stability of the SiQD LEDs based on measurements of the EL intensity as a function of LED operation time (**Figure** **6**a), comparing the results for SiQD LEDs fabricated using three different dispersants; the reproducibility of this data was verified by repeating the measurements (Figure S18, Supporting Information). In particular, the conditions during lifetime measurement were fixed during the long‐term repeat measurements of the four SiQD LEDs as follows: 23 °C temperature, 50% relative humidity, air atmosphere, and the EL intensity was measured in a dark box. A significantly superior half‐life of ≈120 h was estimated for the octane‐dispersed SiQD LEDs by extrapolating EL intensity data (Figure 6a, red circles). In addition, the half‐lives of 12 and 1.76 h were obtained for the toluene‐ and decane‐dispersed SiQD LEDs by interpolating the data in Figure 6a. The 120 h half‐life is 240 times longer than the previous record half‐life for a SiQD LED with a conventional structure (0.5 h),^[^ ^28^ ^]^ and it is three times longer than the value that, to the best of our knowledge, is the record half‐life for an inverted‐structure SiQD LED.^[^ ^28^ ^]^

To explore the mechanism of the degradation of the SiQD LEDs, we tracked the time evolution of the morphology, using optical microscopy, of three different types of SiQD LEDs under an applied voltage. Figure 6b shows optical micrographs of the active areas of the decane‐, toluene‐, and octane‐dispersed SiQD LEDs (top, middle, and bottom, respectively). Many large bubbles were seen in the decane‐ and toluene‐dispersed SiQD LEDs after 5 min and 2 h of operation, respectively, but none were seen in the octane‐dispersed SiQD LEDs, even after 5 h of operation (expanded versions of the images in Figure 6b are shown in Figure S19, Supporting Information). In addition, Figure 6c shows the relationship between the positions of the microbubbles and the two electrodes. From these results, five key points should be highlighted.

First, microbubbles were exclusively formed not at the indium tin oxide (ITO)‐anode edge (i.e., the ITO/PEDOT:PSS/poly‐TPD interface) but at the Al cathode edge (the Al/ZnO/SiQD interface) (Figure 6c). Thus, heat‐induced degradation occurred at the Al/ZnO/SiQD interfaces and became significant for the decane‐dispersed SiQD LEDs. This was because significant Joule heating occurred in this device owing to its high current density (Figure 6b) and low EQE. According to temperature distribution images of the active area captured using a radiation thermometer, the temperature of the decane‐dispersed SiQD LED increased from 23 to 30 °C in 1 min, whereas the temperature change was negligible for the octane‐dispersed SiQD (Figure S20, Supporting Information). In addition, the decane‐dispersed SiQD LEDs featured pinholes (Figure S16, Supporting Information), which resulted in greater carrier densities and Joule heating. In fact, the highest leakage ratio was observed for the decane‐based SiQD LED (Figure S17, Supporting Information).

Second, the microbubble size increased during the period in which the voltage was applied (Figure 6b; Figure S21c, Supporting Information). In other words, Joule heating resulted in progressively increasing microbubble sizes. Similarly, bubble formation at the electrode attributed to the decomposition of organic materials,^[^ ^81^ ^]^ adsorbed moisture,^[^ ^81^ ^]^ polymer materials,^[^ ^82^ ^]^ gas evolution,^[^ ^82^ ^]^ and/or water evaporation^[^ ^83^ ^]^ has been observed in organic LEDs as a result of Joule heating.

Third, PL maps of the active area of the SiQD LEDs acquired after a voltage had been applied (Figure S21b, Supporting Information) revealed that the PL intensity in the microbubble region was reduced. Therefore, the features observed to resemble bubbles were confirmed to be bubbles (void structures). Based on the PL intensity depletion as measured via PL microscopy (Figure S21, Supporting Information), these void structures were formed in the SiQD layer of the SiQD LED.

Fourth, the voids form owing to the vaporization of small amounts of solvent left in the SiQD LEDs because the boiling points of the solvents are lower than those of the solid layer materials. As mentioned earlier, in Figure 6b many voids can be seen to emerge at the Al cathode edge (the Al/ZnO/SiQD interface), and hence it is plausible that vaporization of the solvent (ethanol) used to form the ZnO ETL is the origin of the voids.

Fifth, we discovered that LED degradation was suppressed when the ZnO ETL preparation procedure of the octane‐dispersed LED was modified (Figure 6d). Specifically, for the ZnO ETL preparation process (the last process in Figure 2a), the annealing temperature and time were increased from 100 to 120 °C and from 15 to 30 min, respectively, which may help to eliminate additional residual ethanol from the ZnO ETL. Note that the obtained *T*_70_ value was extended from 80 h (Figure 6a) to 220 h (Figure 6d), which is 8.5 times longer than the record value for an inverted‐structure SiQD LED and 733 times longer than the record value for a conventional‐structure SiQD LED.^[^ ^28^ ^]^ Therefore, the removal of residual ethanol from the ETL was very important for the stability of the SiQD LED as it prevented bubble formation near the Al cathode edge.

Based on these five findings, we conclude that microbubbles with void structures were formed and grew in the inefficient decane‐dispersed SiQD LEDs owing to the fact that the current density was high. Ultimately, the bubbles mechanically destroyed the SiQD layers, resulting in EL decay. In addition, the observed microbubble sizes (≈50 μm) were significantly larger than the total thicknesses of the films in the SiQD LEDs (<0.3 μm), and hence the microbubbles entirely destroyed the layered structure of the LEDs. However, degradation and microbubble formation were significantly reduced in the octane‐dispersed SiQD LEDs. This was because the octane‐dispersed SiQD LEDs had the highest EQE, indicating that the conversion of electron–hole pairs to photons was highly efficient and occurred with minimal Joule heating. In fact, we observed only a few microbubbles in the octane‐dispersed SiQD LED, even in expanded images acquired after 5 h of operation (Figure S19, Supporting Information). These findings indicate that solvent engineering is critical for developing stable SiQD LEDs in which efficient photon generation via electron–hole recombination occurs with minimal Joule heating.

## 3 Conclusion

We synthesized hydrogen‐terminated SiQDs using a cost‐effective HSQ polymer method followed by surface termination with 1‐dodecene via thermal hydrosilylation. The synthesized dodecyl‐terminated SiQDs exhibited a PLQY of 63% in the far‐red (peak PL wavelength: 745 nm). Dispersing these SiQDs in four different solvents (toluene, chloroform, octane, and decane), we fabricated a total of 27 SiQD LEDs with center EL wavelengths in the far‐red region of the visible spectrum (*λ*_EL_ = 750 nm) via solution processing. The performance of the 27 SiQD LEDs was stochastically investigated, and this first study on solvent engineering for SiQD LED fabrication revealed key factors governing the efficiency and stability of the SiQD LEDs. Thus, the light, nontoxic element Si and solvent engineering were used to break four QD LED performance records—for efficiency, luminance, voltage, and lifetime—and the mechanisms underlying these performance improvements were unveiled.

The maximum EQE for the octane‐dispersed SiQD LEDs was 16.5%, which is a new record for SiQD LEDs, to the best of our knowledge. Similarly, the luminance we measured was higher than any value previously recorded for a SiQD LED, and our value was obtained at a voltage five times lower than that used to obtain the previous record. This luminance is comparable to or greater than those of state‐of‐the‐art perovskite QD LEDs emitting in the far‐red. The success in fabricating efficient, bright SiQD LEDs from dodecyl‐terminated SiQDs was attributed to the choice of solvent for the SiQD dispersion. The high wettability and low vapor pressure of octane resulted in the formation of smooth SiQD films owing to the minimization of SiQD aggregation on the poly‐TPD HTL, pinhole formation in the ZnO ETL, and the leakage current of the LEDs.

The EL operation lifetime of the octane‐dispersed SiQD LED was demonstrated to be 733 times higher than the previous record for a SiQD LED with a conventional structure and 8.5 times higher than the previous record for an inverted‐structure SiQD LED. The operational lifetimes of the SiQD LEDs were found to be inversely proportional to the number of microbubbles generated at the Al cathode edge by Joule heating during the application of a voltage that vaporized residual solvent trapped in the ZnO ETL. In fact, no microbubbles were observed for the octane‐dispersed SiQD LEDs because in these samples, which had the highest EQE, very little Joule heating occurred. In addition, further annealing of the ZnO layer to remove residual ethanol extended the *T*_70_ lifetime of the octane‐dispersed SiQD LED from 80 to 220 h.

In summary, this is the first time that solvent engineering has been utilized for SiQD LED development, and it proved to be a very simple, cost‐effective protocol that yielded significantly enhanced SiQD LED performance and operational lifetime. In particular, we found that the octane was the best SiQD dispersant to fabricate our record‐breaking far‐red SiQD LEDs. Such efficient and stable far‐red SiQD LEDs should prove useful as nontoxic light sources to accelerate the growth of fruit and vegetables in green houses. These LED sources could also find application in photodynamic therapy, where far‐red light is used to kill tumors via singlet oxygen generation.

As a result of this study, new avenues are available for the near‐future development of efficient and stable SiQD LEDs with different emission wavelengths, including those in the blue, green, red, far‐red, and near‐IR (800–1000 nm). Although using Si materials with their near‐IR bandgap energy of 1.1 eV to produce IR and far‐IR SiQD LEDs is not straightforward, it could be possible using IR^[^ ^84^ ^]^ and far‐IR^[^ ^85^, ^86^, ^87^ ^]^ emissions from a dopant state, located between the valence band minimum and conduction band maximum, in highly doped SiQDs. For QD synthesis, the light, earth‐abundant element silicon is an excellent alternative to toxic elements and costly precious metals, which have negative human health and environmental impacts. Hence, SiQD LED advancement, by means of solvent engineering among other strategies, is of enormous importance in addressing the grand challenge of designing next‐generation QD LEDs and QD materials for biomedical, display, and solid‐state lighting materials.

## 4 Experimental Section

The methods used to synthesize the SiQDs and their precursors (including the synthesis of the HSQ polymer, the synthesis of the hydrogen‐terminated SiQDs [H‐SiQDs] from the HSQ polymer, and the conversion of the H‐SiQDs into dodecyl‐terminated SiQDs), the characterization of the SiQDs, the SiQD LED fabrication methods, the methods used to characterize the SiQD LEDs, and details of the candela‐unit luminance calculations are described in Section S1–S5, Supporting Information, respectively.

## Conflict of Interest

The authors declare no conflict of interest.

## Author Contributions

**Li Wang**: conceptualization (lead); data curation (lead); formal analysis (lead); funding acquisition (supporting); investigation (lead); methodology (lead); validation (lead); visualization (lead); writing—original draft (supporting); and writing—review and editing (equal). **Yuto Wada**: formal analysis (supporting); investigation (supporting); methodology (supporting); and writing—review and editing (supporting). **Honoka Ueda**: conceptualization (equal); formal analysis (supporting); investigation (equal); methodology (equal); and writing—review and editing (supporting). **Temmaru Hirota**: formal analysis (lead); investigation (lead); methodology (lead); and writing—review and editing (supporting). **Kota Sumida**: investigation (supporting); methodology (supporting); writing—review and editing (supporting). **Yuito Oba**: formal analysis (supporting); investigation (supporting); methodology (supporting); and writing—review and editing (supporting). **Ken‐ichi Saitow**: conceptualization (lead); formal analysis (supporting); funding acquisition (lead); investigation (lead); methodology (lead); project administration (lead); resources (lead); supervision (lead); validation (lead); visualization (supporting); writing—original draft (lead); and writing—review and editing (lead). **Li Wang** and **Ken-ichi Saitow** contributed equally to this work.

## Supporting information

Supplementary Material

## Data Availability

The data that support the findings of this study are available in the supplementary material of this article.
