# Supplementary material for: Record‐Breaking Far‐Red Silicon Quantum Dots LEDs Enabled by Solvent Engineering: Toward Superseding Perovskite Quantum Dots
Source: Small Sci. 2025 Apr 16;5(6):2400647. doi: 10.1002/smsc.202400647 (PMC12168609; doi:10.1002/smsc.202400647)
Supplement: Supplementary file 1 — Supplementary Material [file SMSC-5-2400647-s001.pdf]

Supporting Information

Record-Breaking Far-Red Silicon Quantum Dot LEDs Enabled by Solvent Engineering: Toward Superseding Perovskite Quantum Dots

*Li Wang, Yuto Wada, Honoka Ueda, Temmaru Hirota, Kota Sumida, Yuito Oba, and Ken-ichi Saitow**

Table of contents

S1. Synthesis of Dodecyl-Terminated SiQDs

S1.1. HSQ Polymer Synthesis

S1.2. Synthesis of H-SiQDs from HSQ Polymer

S1.3. Conversion of H-SiQDs into Dodecyl-Terminated SiQDs

S2. Characterization of SiQDs

S3. SiQD LED Fabrication

S4. Characterization of SiQD LEDs

S5. Candela-Unit Luminance Calculations

S6. Supplementary Data

S7. References

S1. Synthesis of Dodecyl-Terminated SiQDs

The synthesis methods employed for the preparation of the HSQ polymer, hydrogen-terminated SiQDs (H-SiQDs), and dodecyl-terminated SiQDs were adapted from our previous study[1–4] with certain modifications to the procedures, as outlined below.

S1.1. HSQ Polymer Synthesis

Trichlorosilane ($HSiCl_3$, 4.5 mL, 0.045 mol) (Nacalai Tesque, Inc., Japan) was gradually introduced to glyme (1,2-dimethoxyethane, 40 mL) (Tokyo Chemical Industry Co., Ltd) in a flask equipped with a magnetic stirrer and immersed in an ice–water bath (2 °C). Subsequently, distilled water was swiftly introduced into the flask containing the well-stirred $HSiCl_3$/solvent mixture. The resulting mixture was stirred for 2 h to complete the dehydration–condensation reaction and synthesize the HSQ polymer. Thereafter, the HSQ polymer was collected as a white precipitate by vacuum filtration followed repeated rinsing with glyme. The polymer was dried on a hot plate at 80 °C for 5 h and then in a vacuum oven at 80 °C for 2 h.

S1.2. Synthesis of H-SiQDs from HSQ Polymer

The HSQ polymer (1 g) was pyrolyzed by placing it in a quartz boat that was subsequently inserted into a tube furnace. It was then pyrolyzed at 1100 °C under a continuous gas flow (95% Ar, 5% H_2; flux rate, 200 mL min^{-1}) for 1 h. Specifically, the sample was heated from 25 to 1100 °C over 2.5 h and then maintained at 1100 °C for a further hour. Finally, the product was cooled from 1100 to 25 °C over a period of 7 to 10 h. Si/SiO_2 matrices (300 mg) were ground from the cooled product using a mortar and pestle. To obtain H-SiQDs, the ground powder was etched in a perfluoroalkoxy alkane (PFA) beaker with a mixture of hydrofluoric acid (HF) (13 mL, 48% w/w Sigma-Aldrich) and hydrochloric acid (HCl) (2 mL, 25% w/w, Sigma-Aldrich) in the dark for 6 h, after which the H-SiQDs were extracted with toluene (15 mL). The H-SiQDs dispersed in toluene were transferred to a polytetrafluoroethylene (PTFE) centrifuge tube and centrifuged at 7800 rpm for 20 min. Subsequently, a molecular sieve (4A, Nacalai Tesque, Inc.) was introduced to the tube to eliminate residual moisture from the toluene. The dispersion of H-SiQDs in dried toluene was subsequently transferred to another tube and centrifuged at 7800 rpm for 20 min.

S1.3. Conversion of H-SiQDs into Dodecyl-Terminated SiQDs

For the thermal hydrosilylation reaction, 1-dodecene and mesitylene were utilized, as well as an H-SiQD dispersion prepared as described in the previous section. First, the toluene was

carefully removed from the H-SiQD dispersion using a pipette. Next, 1-dodecene and mesitylene were added to the tube containing the H-SiQD sediment and the suspension was transferred into a glass flask and subjected to a vacuum for 30 s; this was repeated three times, before the H-SiQDs were refluxed at 130 °C for 2 h under an Ar atmosphere. During this process, the solution became transparent as the reaction progressed. Residual solvent was removed by rotary evaporation. The dodecyl-terminated SiQDs were further purified by means of washing with a solvent (toluene) and then an antisolvent (methanol/ethanol). Finally, the obtained dodecyl-terminated SiQDs were redissolved in 1 mL of toluene and then filtered using a PTFE cartridge filter with a pore size of 0.45 μm.

S2. Characterization of SiQDs

Although almost all the characterization procedures used in this study were based on previously reported methods,[1–6] several aspects of these methods were modified.

The structures and optical properties of the dodecyl-terminated SiQDs were characterized using various experimental methods. The sizes of the dodecyl-terminated SiQDs were measured using transmission electron microscopy (TEM) (JEM-2011, JEOL). Samples were prepared for TEM measurements from a toluene dispersion of the dodecyl-terminated SiQDs. Specifically, the dodecyl-terminated SiQDs in toluene (10 μL, 0.25 mg mL^{-1}) were dropped onto a TEM grid (U1017-5NM, EM, Japan) and allowed to dry for 24 h in a vacuum oven.

The PL and PLE characteristics of the SiQDs dispersed in chloroform were analyzed using a commercial fluorescence spectroscopy instrument (FluoroMax-4, Horiba, Japan). The chloroform dispersion was selected for these measurements in order to obtain the most accurate SiQD characterization results as the absorption of this solvent in the UV is very weak. The UV–vis absorption spectrum of the colloidal SiQDs was measured using a commercial instrument (Spectrophotometer V-660, Jasco).

To determine the crystallinity of the SiQDs, XRD measurements were performed using Cu Kα radiation (40 kV, 50 mA; Smart lab, Rigaku, Japan). In this experiment, a custom-designed nonreflecting silicon sample-holder plate was used to ensure that the signal was background-free.

Raman spectroscopic measurements were conducted using a commercial instrument (HR-800, Horiba, Japan) at an excitation wavelength of 488 nm. To prepare for the Raman acquisitions, the colloidal SiQD solution was dropped onto a stainless-steel plate and the

resultant thin film of SiQDs was used as the sample for the measurements. The integrated Raman intensities were used to compute the crystalline fraction f_c; $f_c = 1/(1 + \gamma I_a^r)$, where I_a^r is the relative Raman intensity for the bands, obtained by dividing the integrated intensity of the amorphous band (I_a) by that of the nanocrystalline band (I_c), and γ is the relative Raman scattering cross section ($\gamma = 0.178$ for a 3-nm Si nanocrystal). This method was also used in our previous studies.[1,4]

Fourier-transform infrared (FTIR) spectra of the SiQDs were measured using the attenuated total reflectance (ATR) method (FT/IR-4200, Jasco). The sample was prepared by dropping the SiQD dispersion (SiQDs in toluene, 10 μL) onto a zinc selenide ATR prism (ATR PRO450-S, Jasco). After drying by blowing room-temperature air onto the prism, FTIR spectra were acquired. The relative amounts of the Si–H, Si–O–Si, and Si–dodecyl functional groups in the dodecyl-SiQDs were determined by analyzing the IR spectra, considering the absorption cross sections. Briefly, the Si–H coverage [based on a sum of the $Si–H_1$, $Si–H_2$, and $Si–H_3$ band intensities (see Figure S3)] was obtained as the relative amount (%) of Si–H as follows: $R_{Si–H} = 100 \times n_{Si–H} / (n_{Si–H} + n_{Si–O–Si} + n_{Si–C})$, where $n_{Si–H} = (1/\langle\tilde{v}\rangle d)\, \sigma^{-1} \int A(\tilde{v})_{Si–H} d\tilde{v}$, $n_{Si–O–Si} = (1/\langle\tilde{v}\rangle d)\, \sigma^{-1} \int A(\tilde{v})_{Si–O–Si} d\tilde{v}$, and $n_{Si–C} = (1/\langle\tilde{v}\rangle d)\, \sigma^{-1} \int A(\tilde{v})_{Si–C} d\tilde{v}$. [The quantities $n_{Si–H}$, $n_{Si–O–Si}$, and $n_{Si–C}$ are the number density of the Si–H, Si–O–Si, and Si–dodecyl functional groups, respectively; $\langle\tilde{v}\rangle$ is the peak wavenumber of the relevant absorption band; d is the optical path length used for the FTIR measurements; s^{-1} is the inverse absorption cross section; $A(\tilde{v})_{Si–H}$, $A(\tilde{v})_{Si–O–Si}$, and $A(\tilde{v})_{Si–C}$ are the absorbances of the Si–H, Si–O–Si, and Si–dodecyl bands, respectively; and $\tilde{v}$ is the wavenumber. However, note that d can be eliminated from this expression because the Si–H and Si–O–Si IR spectra were measured using an identical sample at the same time.] This calculation method has been described in detail in previous reports.[1,4]

We calculated the energies of the dodecyl-terminated SiQDs using a previously reported method.[1,3–5] The bandgap energy was determined from the energy of the PL band peak as 1.66 eV. The conduction and valence band energies of the dodecyl-terminated SiQDs were calculated using the equations $\Delta VB = 2\Delta CB$ and $E_g = 3\Delta CB + 1.1$ eV. In these equations, ΔVB and ΔCB are the energies of the valence and conduction bands of the SiQDs relative to bulk silicon, and the former and latter were obtained as 0.37 and 0.19 eV, respectively.

S3. SiQD LED Fabrication

A pre-patterned indium-tin-oxide (ITO) glass substrate (Flat ITO, Geomatec Co., Ltd.) was cleaned using a flat panel display detergent solution and then distilled water. The cleaned ITO substrate was placed in a UV-ozone cleaner (PC450, Bioforce) for 15 min and subsequently used as the cathode for the LED. Similar procedures are reported elsewhere.[2,3]

A hole injection layer of poly(3,4-ethylenedioxythiophene) (styrenesulfonate) (PEDOT:PSS) was deposited onto the cleaned ITO glass substrate by spin-coating (500 rpm, 3s; 3000 rpm, 30s) using an aqueous solution (Clevios™ PVPAI 4083, Ossila Ltd) in air. The coated substrate was then annealed at 150 °C in air for 15 min. Subsequently, a poly[*N*,*N*'-bis(4-butylphenyl)-*N*,*N*-bis(phenyl)benzidine] (poly-TPD; American Dye Source, Inc.) hole transport layer (HTL) was spin-coated (500 rpm, 1s; 5000 rpm, 30s) onto the PEDOT:PSS layer from an *o*-dichlorobenzene solution (8 mg mL^{-1}) in an argon-filled glove box (O_2 and H_2O, < 0.1 ppm). The poly-TPD layer was annealed at 180 °C for 1 h in the glove box. An emissive SiQD layer was subsequently deposited by spin-coating (2000 rpm, 30s) using chloroform, decane, octane, and toluene SiQD dispersions (15 mg mL^{-1}), followed by thermal annealing at 70 °C for 30 min in the glove box. An electron transport layer (ETL) was formed by spin-coating, using a ZnO nanoparticle prepared in this study based on a previously reported method dispersion (20 mg mL^{-1}; ethanol:isopropyl alcohol, 3:1 vol/vol),[4] followed by annealing at 100 °C for 15 min in the glove box. A 100-nm aluminum cathode was deposited by thermal evaporation in a vacuum chamber with a shadow mask. Finally, the SiQD LEDs were sealed with epoxy resin in the glove box to prevent air exposure.

S4. Characterization of SiQD LEDs

The current–voltage curves and optical powers of the fabricated SiQD LEDs were characterized using a source meter (2400C, Keithley Instrument Inc.) and optical power sensor (PD300-UV, Ophir), respectively. The EL spectra were measured using a monochromator equipped with a CCD camera (SpectroPro 2300i, Princeton Instrument). The external quantum efficiency (EQE) of the SiQD LEDs was obtained using the equations reported in the Results and Discussion section (Section 2 in the main manuscript). The luminance of the LEDs was measured using a commercial luminance color meter (CS-200, Konica Minolta, Inc.). These characterization protocols were based on those used in previously reported studies.[1,3–5]

We evaluated the EQE based on a previously reported method[1,3,4] using the equation $EQE = (P/h\nu)/(I/e)$, where P is the optical power density (Figure 2f), $h\nu$ is the EL photon energy

at λ_{EL} = 750 nm, I is the current density (Figure 2e), and e is the elementary charge. The obtained SiQD LED EQEs are plotted as a function of the current density in Figure 2g; the EQE values shown were calibrated using the light correction efficiency and solid angle of EL based on a method reported elsewhere.[1,3,4,7,8] Of particular note is the fact that the EQE was high at low current densities, a trend observed in many previous studies on SiQD LEDs.[1,3,4,8–16] This is because a low current density produces a high EQE, as can be seen from the form of the equation for the EQE quoted above. In addition, it has also been recognized that high carrier densities, arising from high current densities, promote nonradiative processes such as Auger recombination.[4,17]

To investigate the mechanisms underlying the observed effectiveness of our solvent engineering approach, SiQDs prepared as chloroform, decane, octane, and toluene dispersions were deposited onto poly-TPD layers via spin-coating, and the resulting films were subsequently evaluated following modified procedures based on those used in previously reported studies.[18–24]

Images of island-like SiQD aggregate structures on the HTL film used for SiQD LED construction were captured using a laser microscope (OLS4000, Olympus, Japan). The SiQD aggregate area of each film (Figure 3) was obtained as the sum of the area of each individual SiQD aggregate in the observation area (1.34 mm × 1.34 mm) divided by the observation area. The areas of the spherical and tail-like aggregates were calculated as circles and rectangles (or triangles), respectively, using lengths measured from the obtained laser microscopy images. PL spectra of island-like SiQD aggregate structures on these HTL films acquired using a confocal spectromicroscope (LabRAM HR-800, Horiba, Japan) with an excitation wavelength of 488 nm (543-AP-A01, Melles Griot, United States), a power of 0.12 mW, and a 100× objective lens with a numerical aperture (NA) of 0.60 (SLMPlan N, Olympus, Japan). For the PL mapping measurements, PL spectra were collected from a 31 μm × 31 μm area of the SiQD film that included regions with bubbles. To construct the maps, the spectra in 961 pixel areas (31 × 31 pixels, with a resolution of 1 μm per pixel) were acquired using a high-precision x–y stage with an interval of 0.1 μm. These characterization protocols were based on those used in previously reported studies.[18–24]

The thickness of the SiQD films at positions corresponding to the spectral measurements was assessed using the same laser microscope, with thickness mapping conducted using an axial (z-axis) resolution of 10 nm. The PL spectra and film thickness were measured at the same locations using confocal and laser microscopy, respectively; the lateral (x–y) positional accuracy was within 500 nm.

To assess the wettability of the poly-TPD HTL film by the SiQD dispersions, contact angle measurements were performed using a contact angle measurement instrument (Dropmaster 700, Kyowa Interface Science Co., Ltd., Japan) and the chloroform, decane, octane, and toluene dispersions of SiQDs.

To investigate the degradation of the SiQD LEDs, using the optical mode of a laser microscope (OLS4000, Olympus), we tracked the time evolution of three different types of SiQD LEDs under an applied voltage of 6 V over the time interval from 5 min to 5 h after the start of LED operation. The monitored area was the entire active area of the SiQD LED (2 mm × 2 mm), and images were captured using ten-fold magnification from the Al cathode side of the LED.

S5. Candela-Unit Luminance Calculations

We discuss the luminance calculations in units of cd m^{-2}. To accurately compare the results of the present study with those of previous studies on SiQD LEDs, the luminance was calculated using two different equations. This is because in several studies on SiQD LEDs,[7,25] an equation was used to compute the luminance (Equation S1) that produced values 2–3 orders of magnitude greater than those obtained using a conventional equation (Equation S2), and therefore the values obtained in the present study using each equation are listed in **Table S3**. Upon comparing the results with experimental luminosity data measured using a commercial luminance measurement instrument (CS-200, Konica Minolta), we concluded that the luminance values calculated using Equation S2 were in good agreement with the measured values obtained for the same SiQD LEDs (Figure S7) and that Equation S1 incorrectly overestimated the luminance. Moreover, compared with all the results in the literature, higher luminosity, 66 cd m^{-2}, was obtained for the decane-dispersed SiQD LED we fabricated in the present study even though we used a voltage (6 V) five times lower than that used in a previous study to generate a similar but slightly lower luminance (58 cd m^{-2} at 30 V);[13] further details of these results are supplied in Table S2. In addition, it should be mentioned that the luminance values obtained in the present study were for EL at redder wavelengths (λ_{EL} = 750 nm, in the far-red) with respect to the EL wavelength observed in the aforementioned previous study (λ_{EL} = 685 nm). Note that far-red light is light with a wavelength in the range of 700–800 nm, close to the wavelength range of near IR light.

In several previous SiQD LED studies, very large luminances of 5,000–20,000 cd m^{-2},[7,25] i.e., 2–3 orders of magnitude higher than those reported in other previous SiQD LED

studies, were reported. As the EL wavelength corresponding to these literature results was in the far-red, and far-red emissions are associated with low luminance owing to the form of the CIE diagram (Figure S8), the reported luminance values were obviously too high to be considered accurate. Therefore, we verified the calculated luminance values by comparing the results obtained using the following two equations with luminances measured using a typical commercial instrument.

First, the luminance calculation method mentioned above and reported elsewhere[7,25] was based on a specific equation (Equation S1), for which the authors cited a Master's dissertation.[26]

$$\text{luminance}\ \left(\mathrm{cd\ m^{-2}}\right) = \frac{K_\mathrm{m} \cdot J \cdot \mathrm{EQE}}{2\pi \cdot S_\mathrm{d}} \int_{380}^{780} I(\lambda) \cdot k(\lambda)\, \mathrm{d}\lambda \qquad \text{(S1)}$$

where K_m is the maximum luminous efficiency (683 lm W^{-1}), $I(\lambda)$ is the relative EL intensity as a function of wavelength (the normalized intensity at each EL wavelength), J is the current density, S_d is the device area, EQE is the external quantum efficiency, and $k(\lambda)$ is the CIE standard photopic efficiency function, i.e., $k(\lambda)$ is the same as the function CIE(λ) reported elsewhere.[7] Using this equation, the values obtained were reported as 19,934[7] and 5,000 cd m^{-2}.[25] Similarly, using Equation S1, the luminance of our SiQD LED was calculated to be 116,822 cd m^{-2}, which is not reasonable for luminance in the far-red.

Next, we calculated the luminance using a conventional equation that is frequently used to calculate the luminance values of QD LEDs (Equation S2, below).[27–29] Equation S2 is quoted in a total of 135 papers [the total number of citations for the original papers[30,31] according to Google Scholar].

$$\text{luminance}\ \left(\mathrm{cd\ m^{-2}}\right) = \frac{\mathrm{EQE} \cdot K_\mathrm{m} \cdot h \cdot J \cdot \int_{380}^{780} I(\lambda) \cdot k(\lambda)\, \mathrm{d}\lambda}{\pi \cdot e \cdot \int_{380}^{780} I(\lambda) \cdot \lambda\, \mathrm{d}\lambda} \qquad \text{(S2)}$$

where h is Planck's constant, c is the speed of light, and e is the elementary charge.[30,31] Using Equation S2, the abovementioned luminances of 19,934[7] and 5,000 cd m^{-2} [25] were recalculated as 6.62 and 10.46 cd m^{-2} (λ_{EL} = 755 and 720 nm, respectively), and the result for the present study was 66 cd m^{-2} (λ_{EL} = 750 nm). Although the differences between Equations S1 and S2 are not discussed here, the reader can compare the forms of the two equations to observe the differences. We also measured the luminance of our SiQD LEDs using a commercial luminance meter (CS-200, Konica Minolta) and compared the result with the calculated values. The experimental values obtained were in good agreement with the values calculated using Equation S2 (Figure S7). In conclusion, Equation S2 is appropriate for luminance calculations, and it has been used in many QD LED studies.[27–29] In contrast, the

significantly larger luminance values reported in previous studies[7,25] were obtained owing to the use of Equation S1, which incorrectly overestimated the luminance.

Finally, we evaluated the abovementioned high luminance of the present far-red SiQD LED (i.e., 66 cd m^{-2} at λ_{EL} = 750 nm) by comparing with other state-of-the-art QD LEDs. As described in section 2.3 of the present paper, the luminance values of state-of-the-art perovskite QD LEDs in the far-red wavelength region (700–800 nm) are much lower than those in visible region; for example, values of 180 cd m^{-2} (λ_{EL} = 735 nm) [32] and < 4.3 cd m^{-2} (λ_{EL} = 772 nm) [33] have been observed. Specifically, for the latter case, the maximum luminance of the perovskite QD LED was reported as 4.3 cd m^{-2} (λ_{EL} = 692 nm), and the luminance (λ_{EL} = 772 nm) resulted in two orders of magnitude smaller than that of 4.3 cd m^{-2} (λ_{EL} = 692 nm),[33] based on the CIE photopic spectral luminous efficiency function (the inset of Figure S8). In addition, other studies on perovskite QD LEDs were surveyed, but we were not able to find the luminance data of the far-red perovskite QD LEDs (λ_{EL} = 700−800 nm), although there are two papers of near far-red perovskite QD LEDs, i.e., 206 cd m^{-2} (λ_{EL} = 698 nm)[34] and 120 cd m^{-2} (λ_{EL} = 693 nm).[35] Furthermore, to the best of our knowledge, LEDs with high luminance in the far-red have not been achieved using Cd based or In-based QDs because the bandgap energies of these materials in the bulk (e.g., CdSe and InP, 1.4–1.8 eV) are significantly greater than that of silicon (1.1 eV). Therefore, it was concluded that the SiQD LEDs fabricated in this study exhibited luminance in the far-red (66 cd m^{-2}, λ_{EL} = 750 nm) is comparable to or higher than those of state-of-the-art QD LEDs.

S6. Supplementary Data

a

b

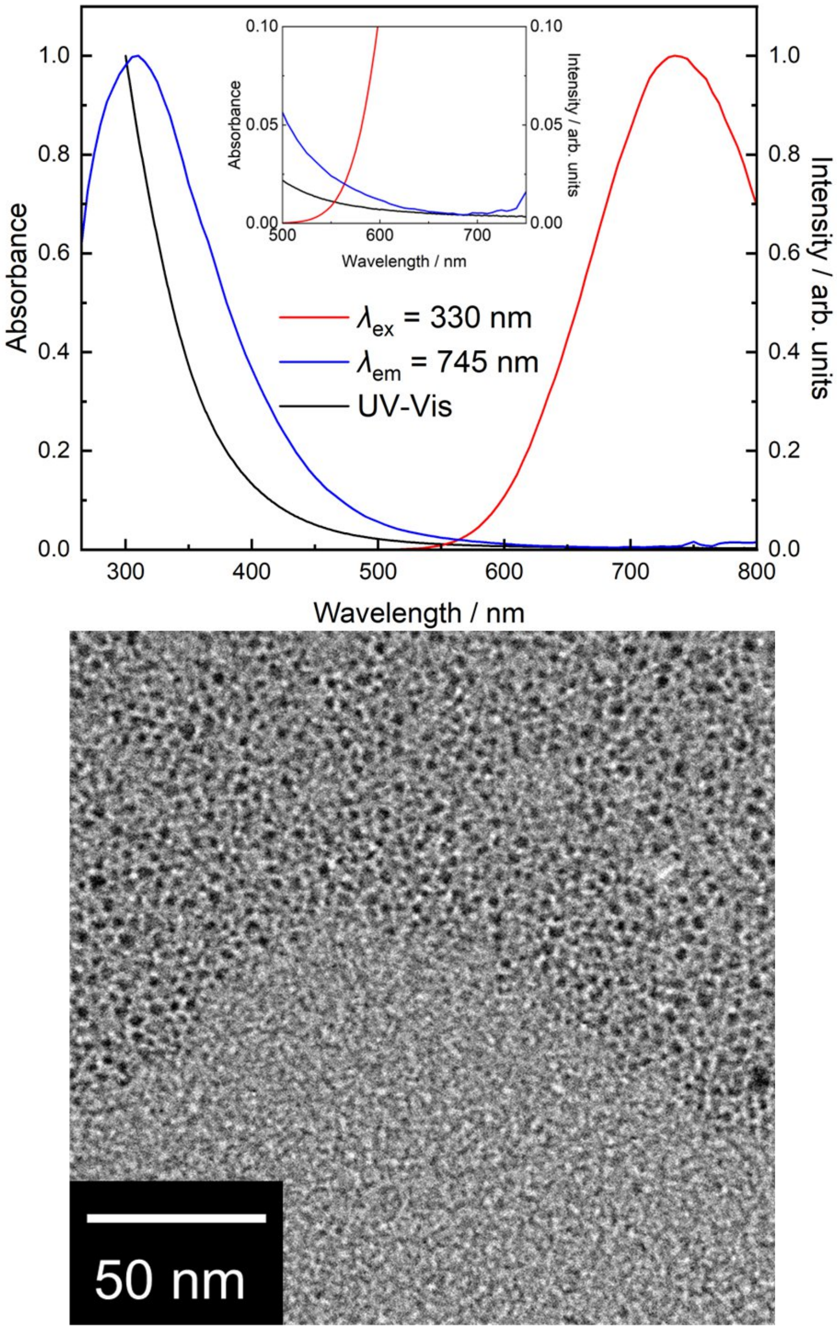

Figure S1. Characterization of dodecyl-terminated SiQDs. a) PL (red line, measured with excitation at 330 nm), PLE (blue line, measured at an emission wavelength of 745 nm), and UV–Vis absorption (black line) spectra. The figure shows the data shown in Figure 1b but with a linear wavelength scale on the horizontal axis. The inset shows expanded data in the wavelength region of 500–750 nm; the minor PLE band at 745 nm is stray excitation light. b) Expanded view of the central area of the TEM image shown in Figure 1c. The upper area, featuring blacker particle-like structures, and lower area, featuring grayer less-defined particle-like structures, correspond to an area of dodecyl-terminated SiQDs and the carbon support film on the TEM grid, respectively.

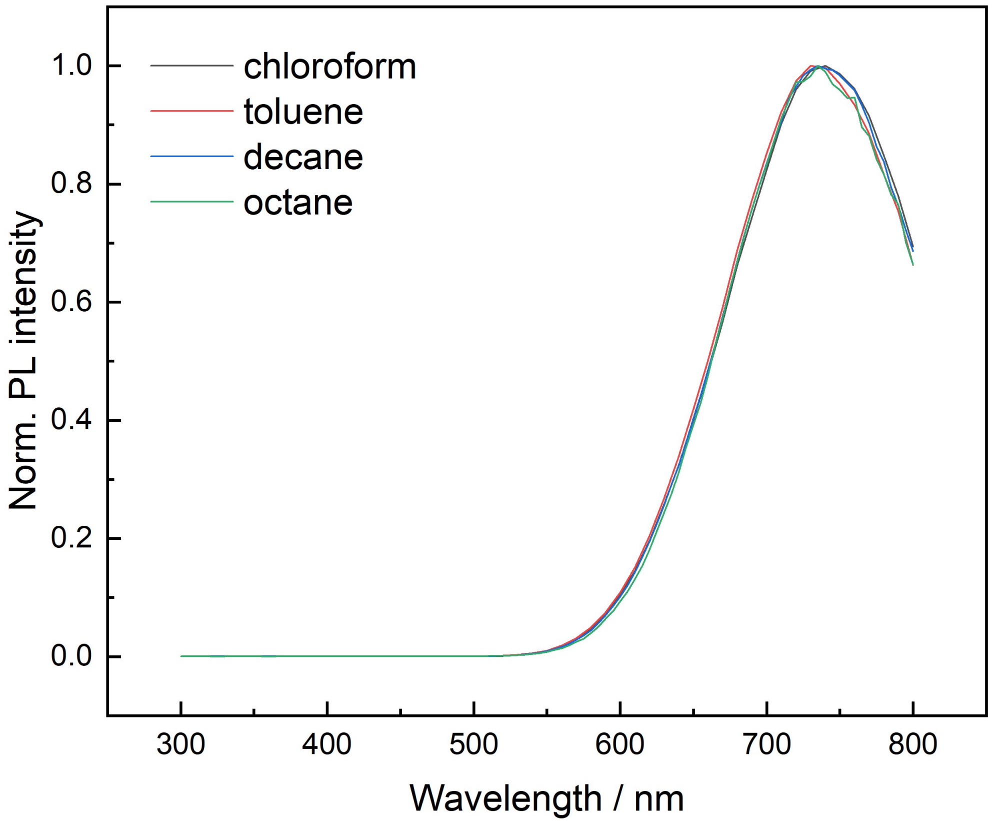

Figure S2. PL spectra of dodecyl-terminated SiQDs dispersed in four solvents. Negligible differences are observed.

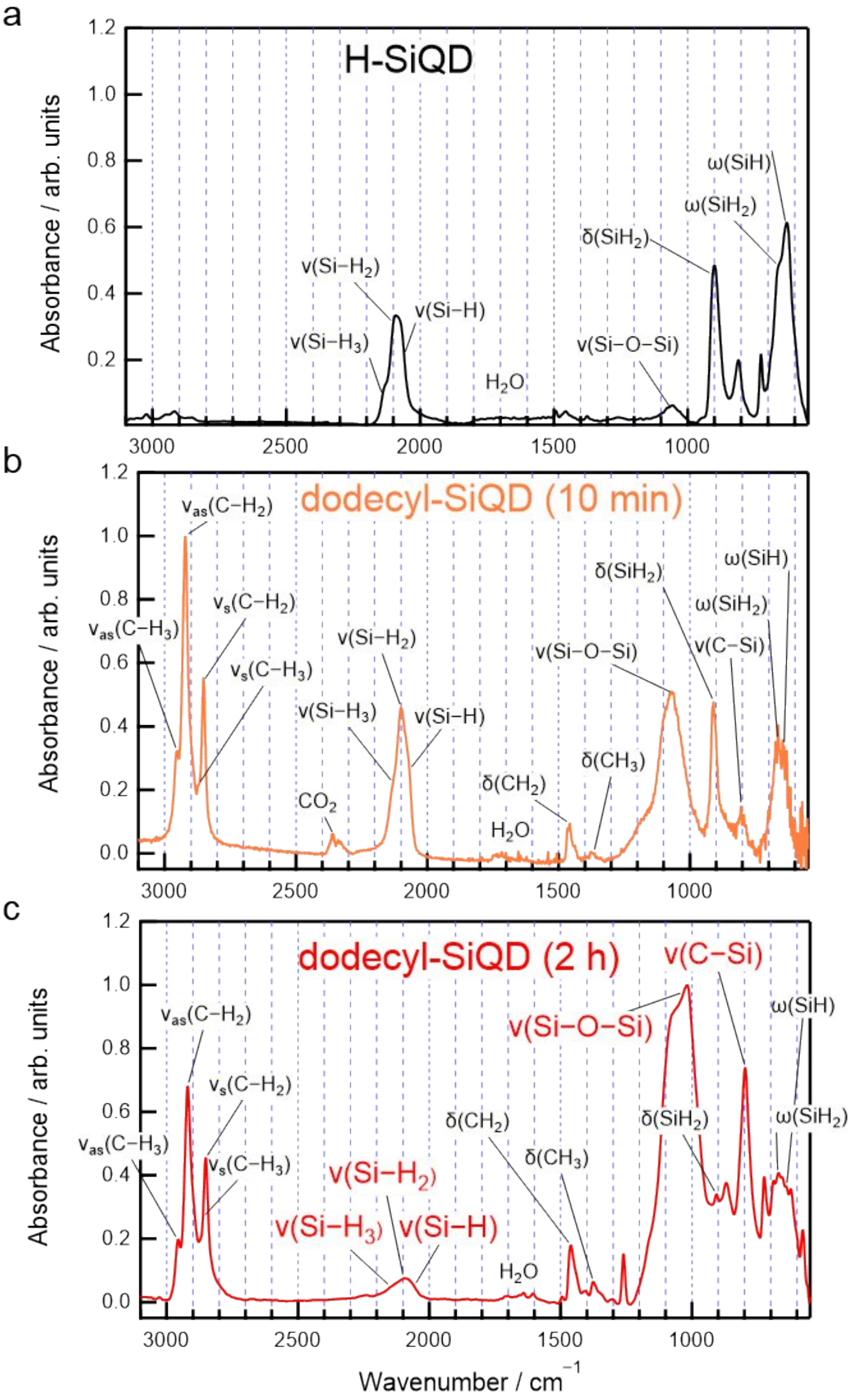

Figure S3. FTIR spectrum of dodecyl-terminated SiQDs. Spectra acquired a) before passivation (spectrum of H-SiQDs), b) after passivation for 10 min, and c) after passivation for 2 h (the conventional passivation time). The coverages of the Si–H, Si–O–Si, and Si–dodecyl functional groups were obtained from the intensities of the bands assigned to the respective modes, which are highlighted by the larger red font for the assignment labels in the lowest panel; an average of five measurements was used to obtain each coverage, and the Si–H coverage was calculated by summing the Si–H_1, Si–H_2, and Si–H_3 band intensities. The symbols ν, δ, and ω are used to denote stretching, scissoring, and wagging modes, respectively. The shoulder of the Si–O–Si band was attributed to the out-of-plane mode. The assignments for the FTIR bands were based on the results of previous studies.[4,36–40] Absorption cross sections were used as reported in the literature.[41–43]

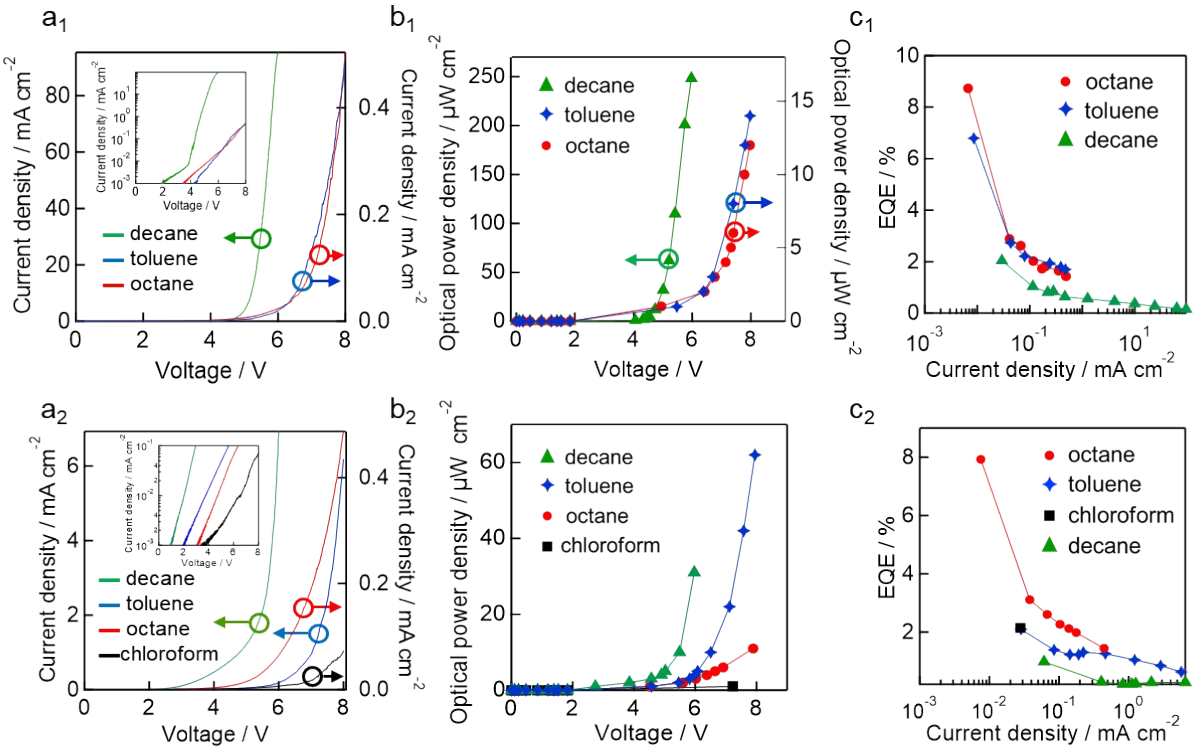

Figure S4. Repeat SiQD LED optoelectronic performance measurements. *I–V–L* data for decane-, octane-, toluene-, and chloroform-dispersed SiQD LEDs. a_1–c_1) Second and a_2–c_2) third repeat experiments performed using different SiQD LEDs to verify the reproducibility. a_1, a_2) *I–V* curves. For clarity, the insets show the same data as in the main panels plotted using log scales for the vertical axes. b_1, b_2) *L–V* curves. c_1, c_2) External quantum efficiencies (EQEs) of SiQD LEDs as functions of current density. The color indicates the solvent used (red: octane; green: decane; blue: toluene; black: chloroform). Note that as the chloroform-dispersed SiQD LED showed very poor performance, optoelectronic performance data was not recorded multiple times in this case.

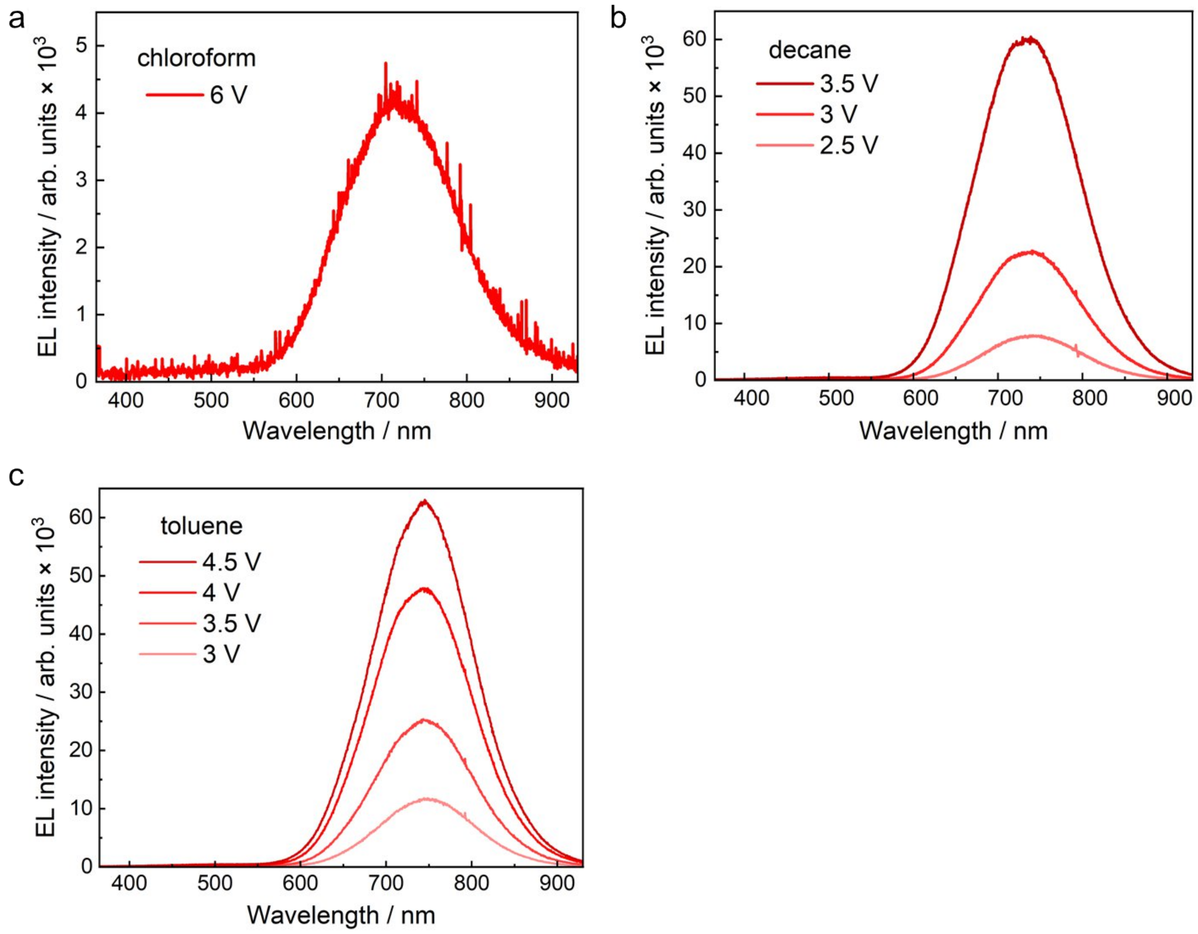

Figure S5. EL spectra of three different SiQD LEDs. Spectra of a) chloroform-, b) decane-, and c) toluene-dispersed SiQD LEDs. The EL intensity in the spectra of the chloroform-dispersed SiQD LEDs, shown in panel a, was very weak, and hence the data was collected at an applied voltage of 6 V.

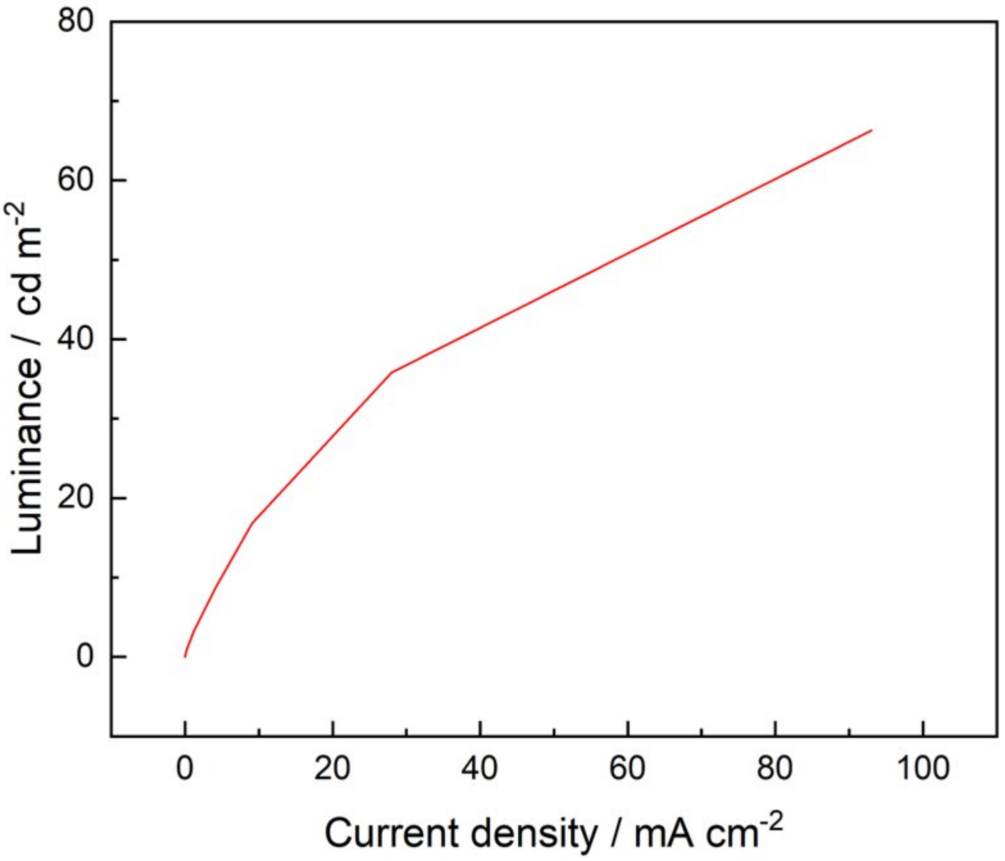

Figure S6. Luminance as a function of current density. The maximum luminance obtained was 66 cd m^{-2}. The details are denoted in Section S5 and Table S2.

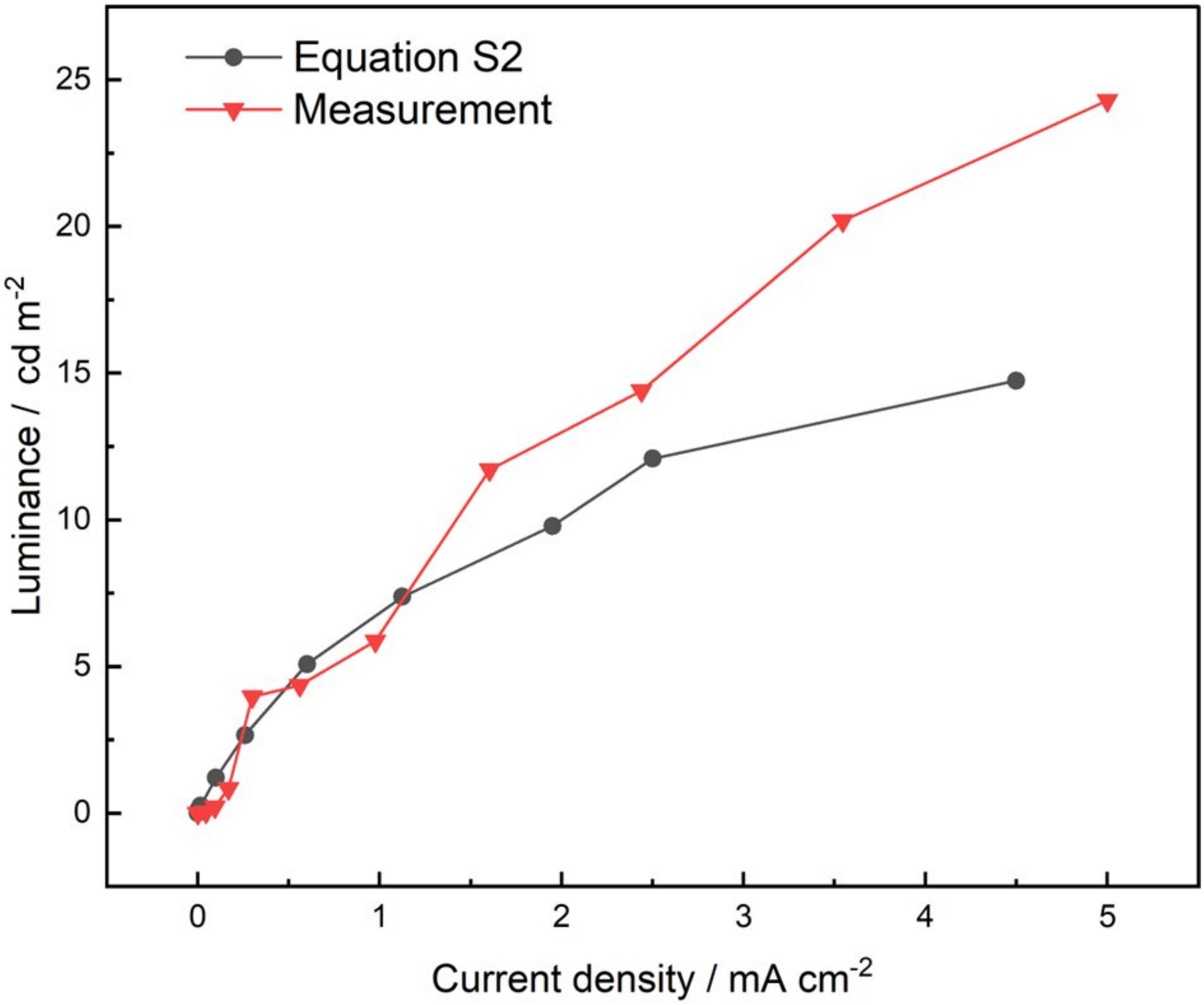

Figure S7. Luminance of octane-dispersed SiQD LED as a function of current density. Black circles and red triangles represent theoretical results calculated using Equation S2 and experimental data, respectively.

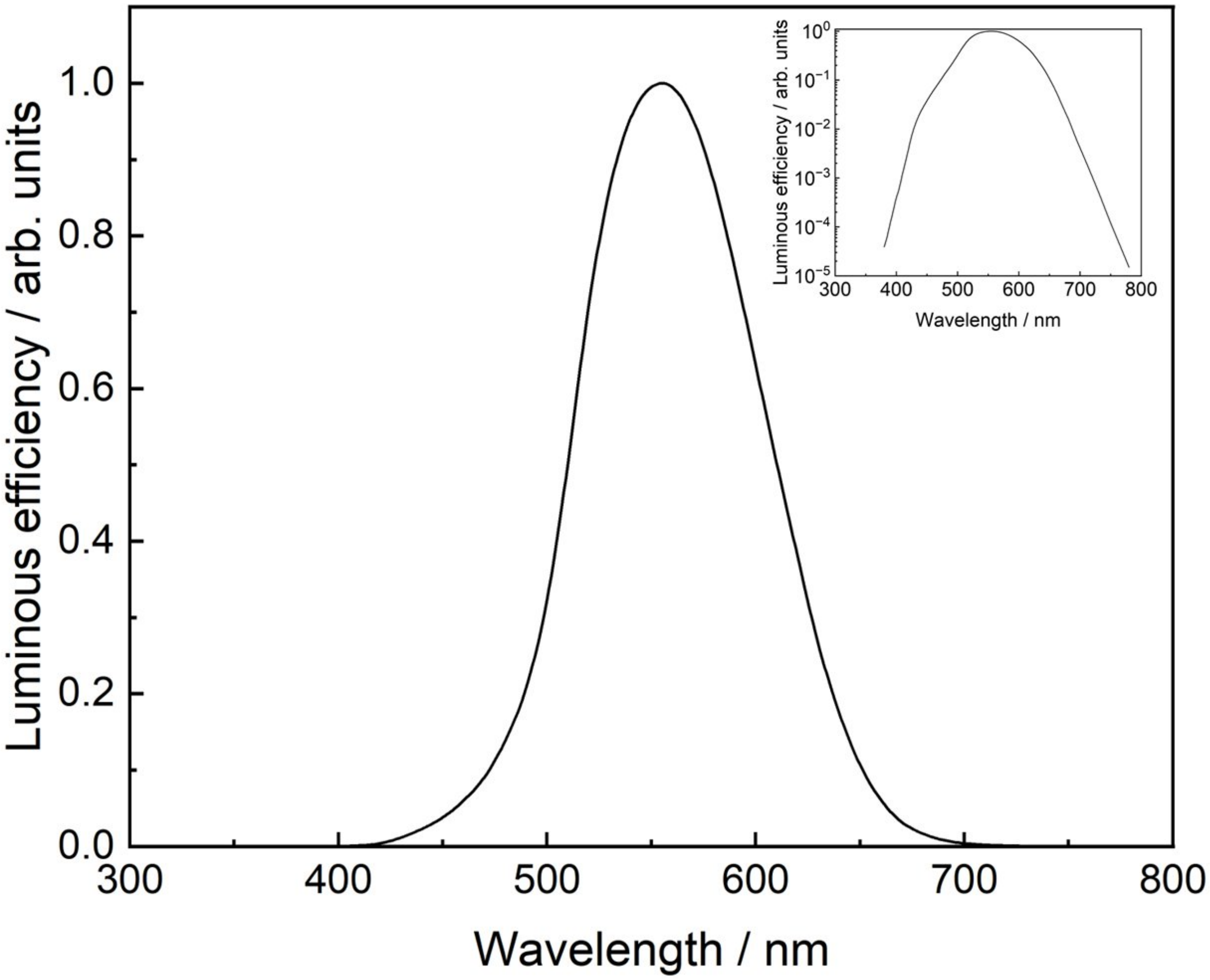

Figure S8. CIE photopic spectral luminous efficiency function. The inset shows the same data plotted using a log scale on the vertical axis. Data reproduced from the website of the International Commission on Illumination.[44]

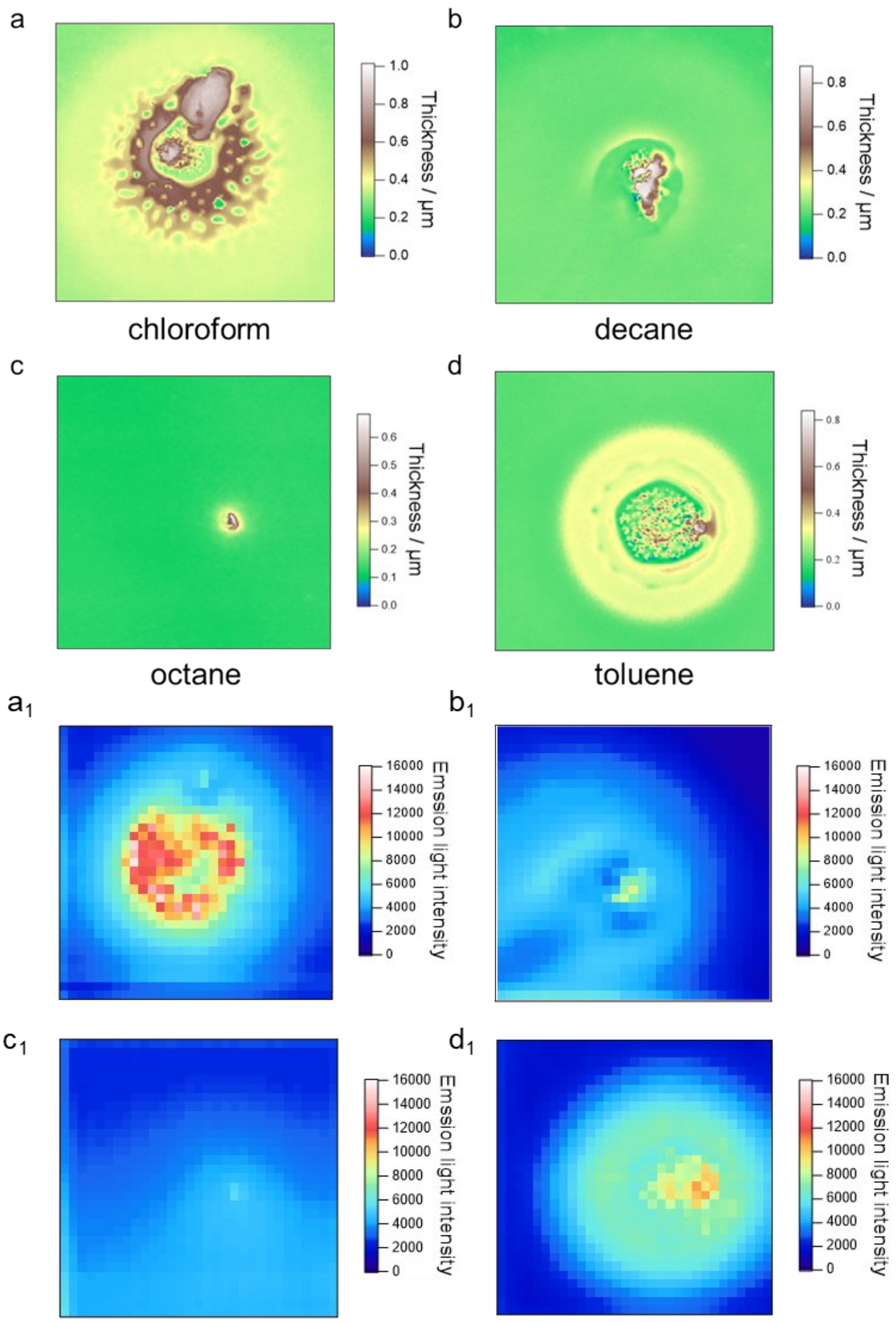

Figure S9. Laser micrographs and PL maps of SiQD LEDs showing island aggregate structures in the SiQD layers formed by spin-coating poly-TPD layers using four dodecyl-terminated SiQD dispersions. a–d) Confocal images showing sample thickness and a_1–d_1) PL maps of island structures at the corresponding positions in the same samples. The PL maps were measured using an excitation wavelength of 488 nm. The PL wavelength used for mapping was 700 nm in each case. Each image shows an area of 31 µm × 31 µm.

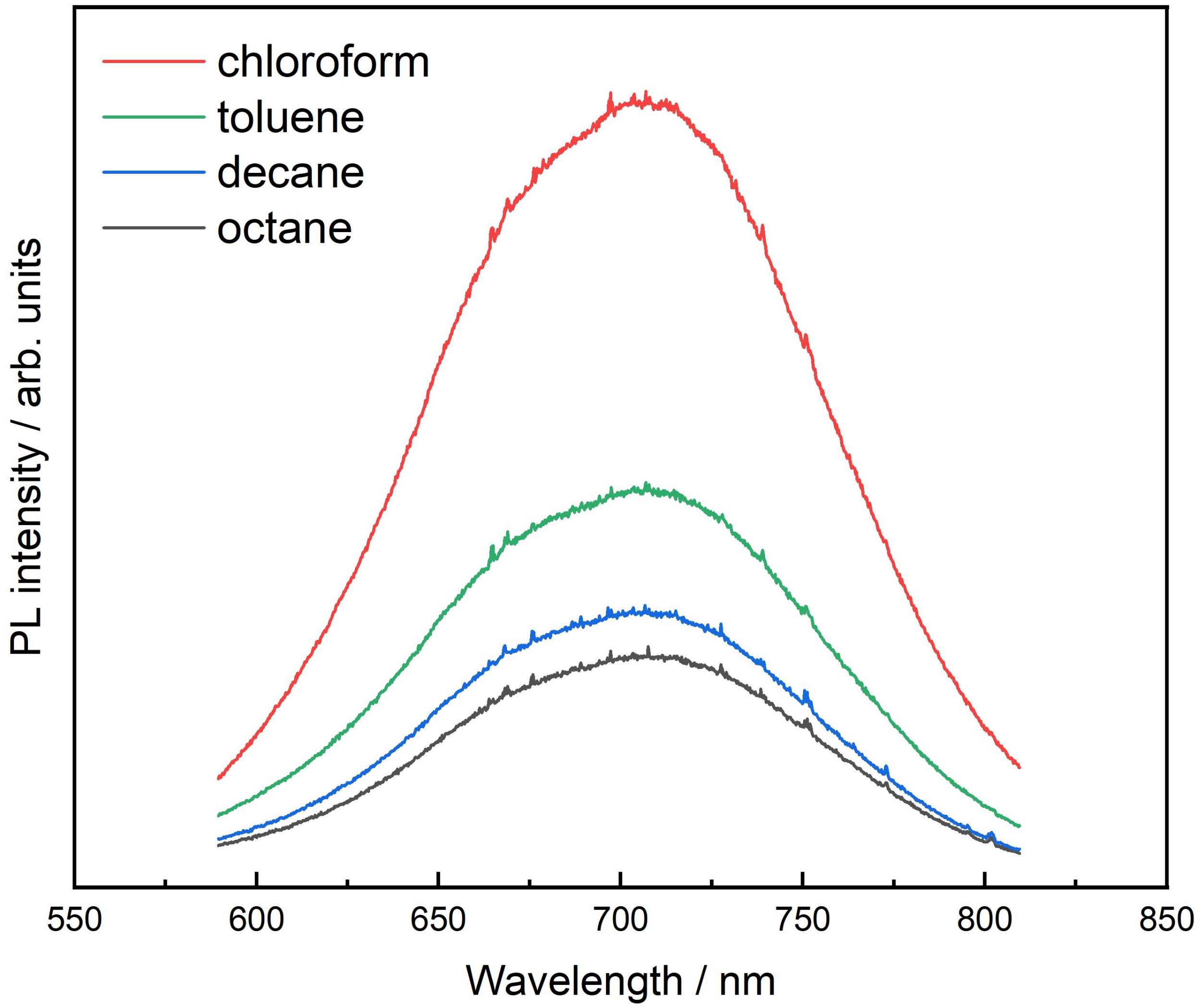

Figure S10. PL spectra of island-like SiQD aggregate structures in films used for SiQD LED construction corresponding to the confocal microscopy images shown in Figure S9. The spectra were acquired under excitation at 488 nm. Each spectrum was obtained at a point in the maps shown in Figure S9 corresponding to one of the islands (SiQD aggregates). The high and low PL intensities of the chloroform- and octane-dispersed SiQD LEDs are owing to the large and small sizes of the SiQD aggregates, respectively.

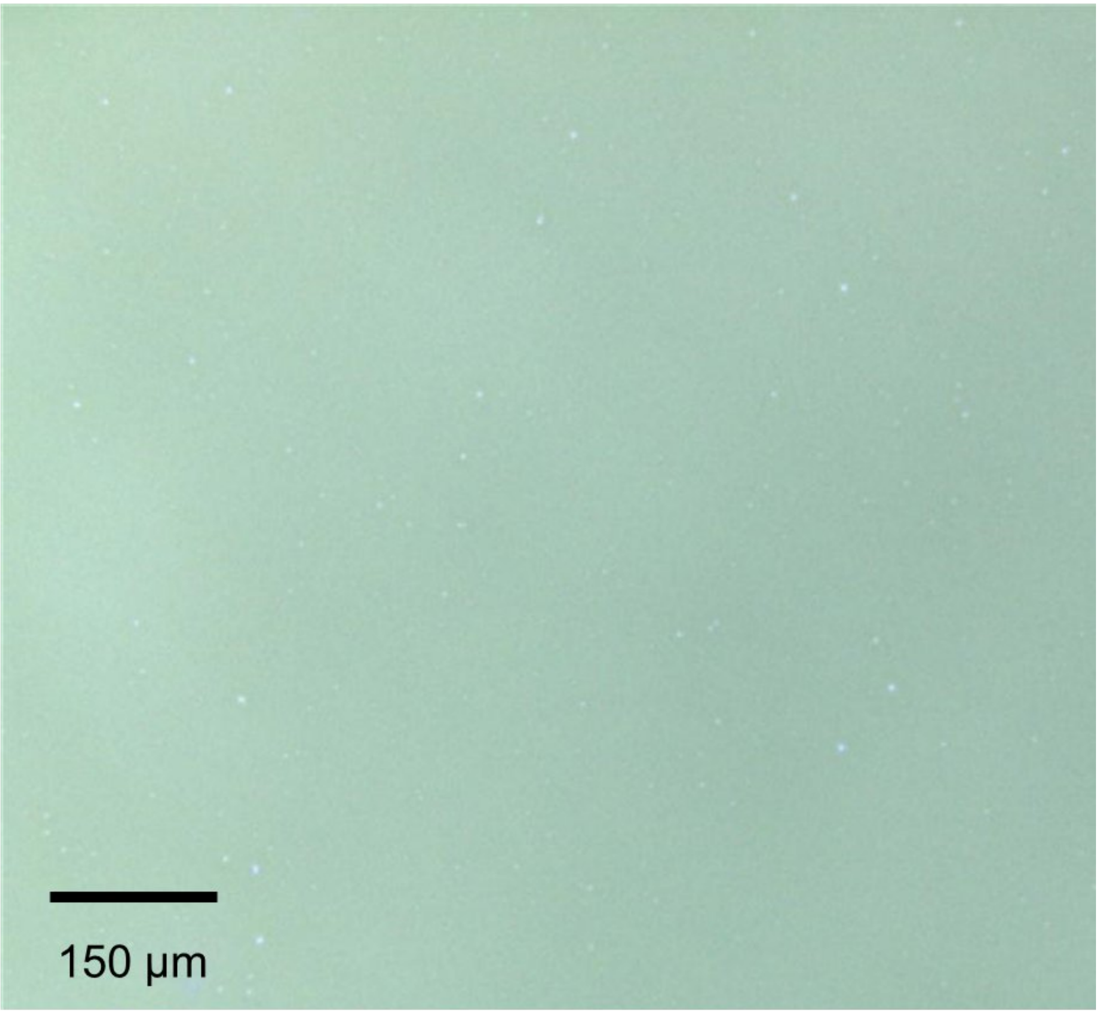

Figure S11. Expanded view of optical micrograph of SiQD layer shown in Figure 3b. The sample was prepared by spin-coating the poly-TPD layer of the LED with a dispersion of SiQDs in octane.

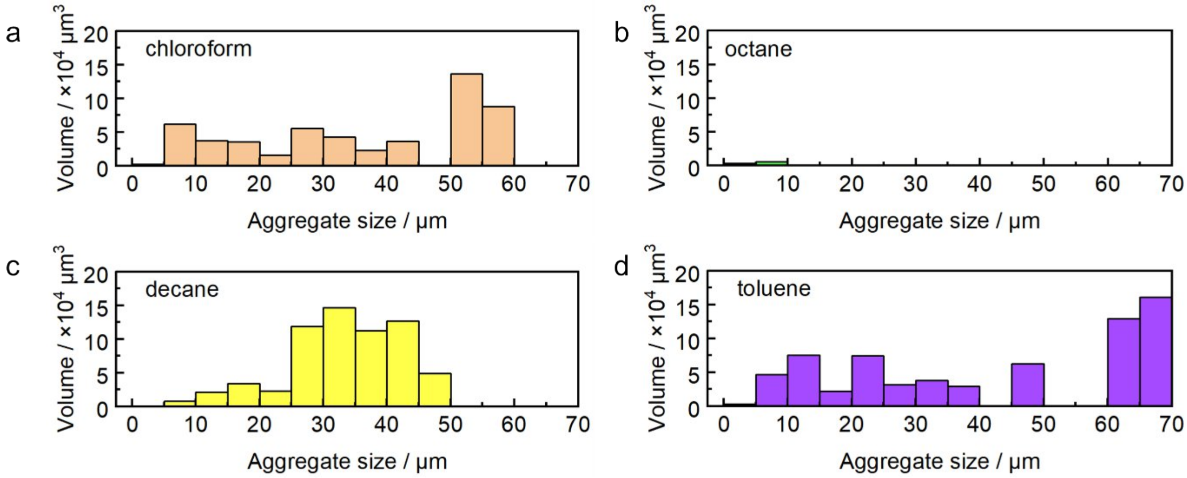

Figure S12. Volumes of SiQD aggregates vs. size. The analysis was conducted using the data shown in Figure 3c.

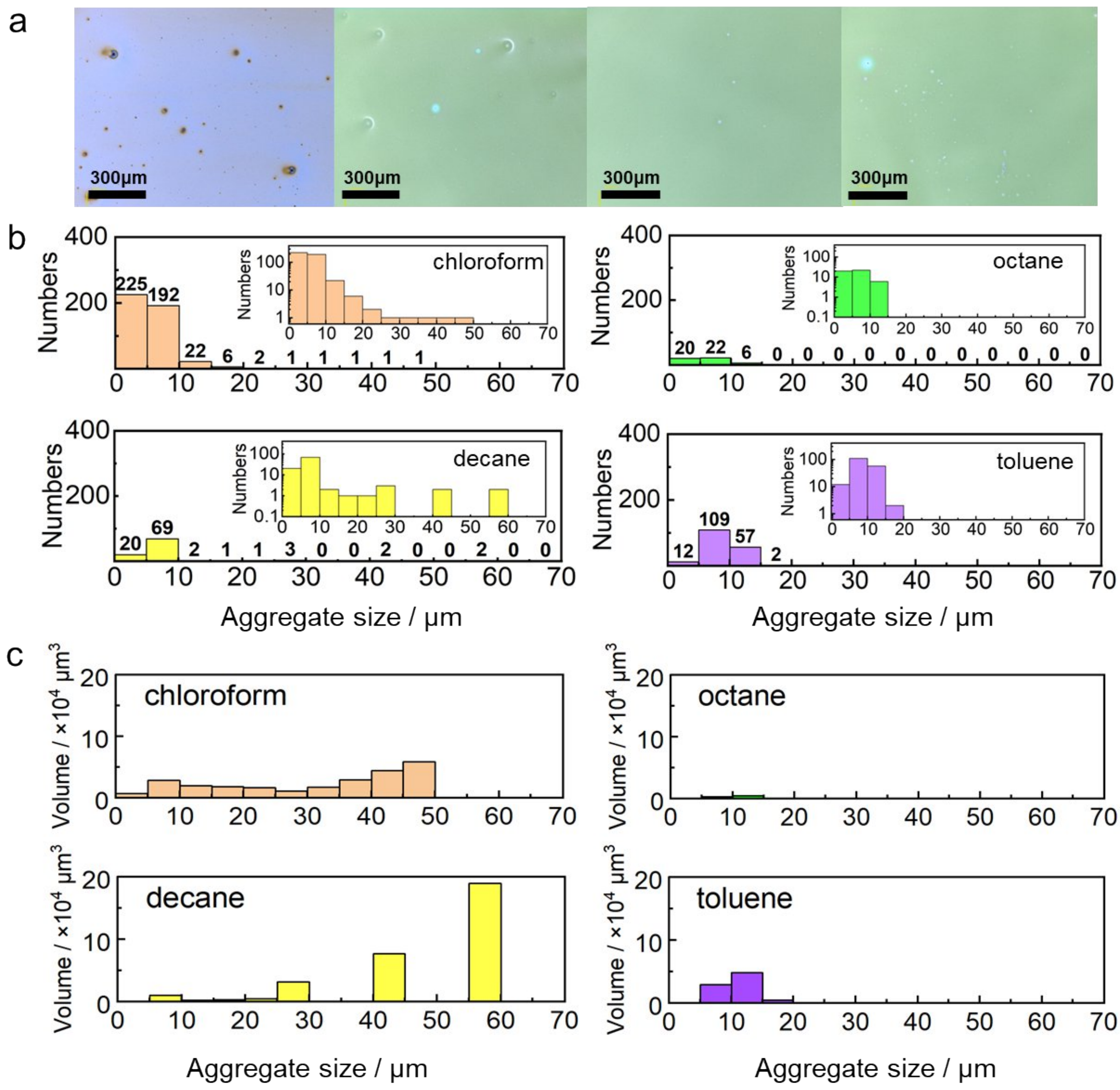

Figure S13. Results of repeat SiQD aggregate imaging measurements and analysis. a) Optical micrographs of SiQD layers prepared by spin-coating the poly-TPD layer of SiQD LED structures using dispersions of SiQDs in the four solvents shown in panel a, respectively. b) Size distributions of the SiQD aggregates obtained via analysis of the images shown in panel b; the insets show the same data plotted using log scales on the vertical axes. c) Volume distributions corresponding to the size distributions shown in panel b.

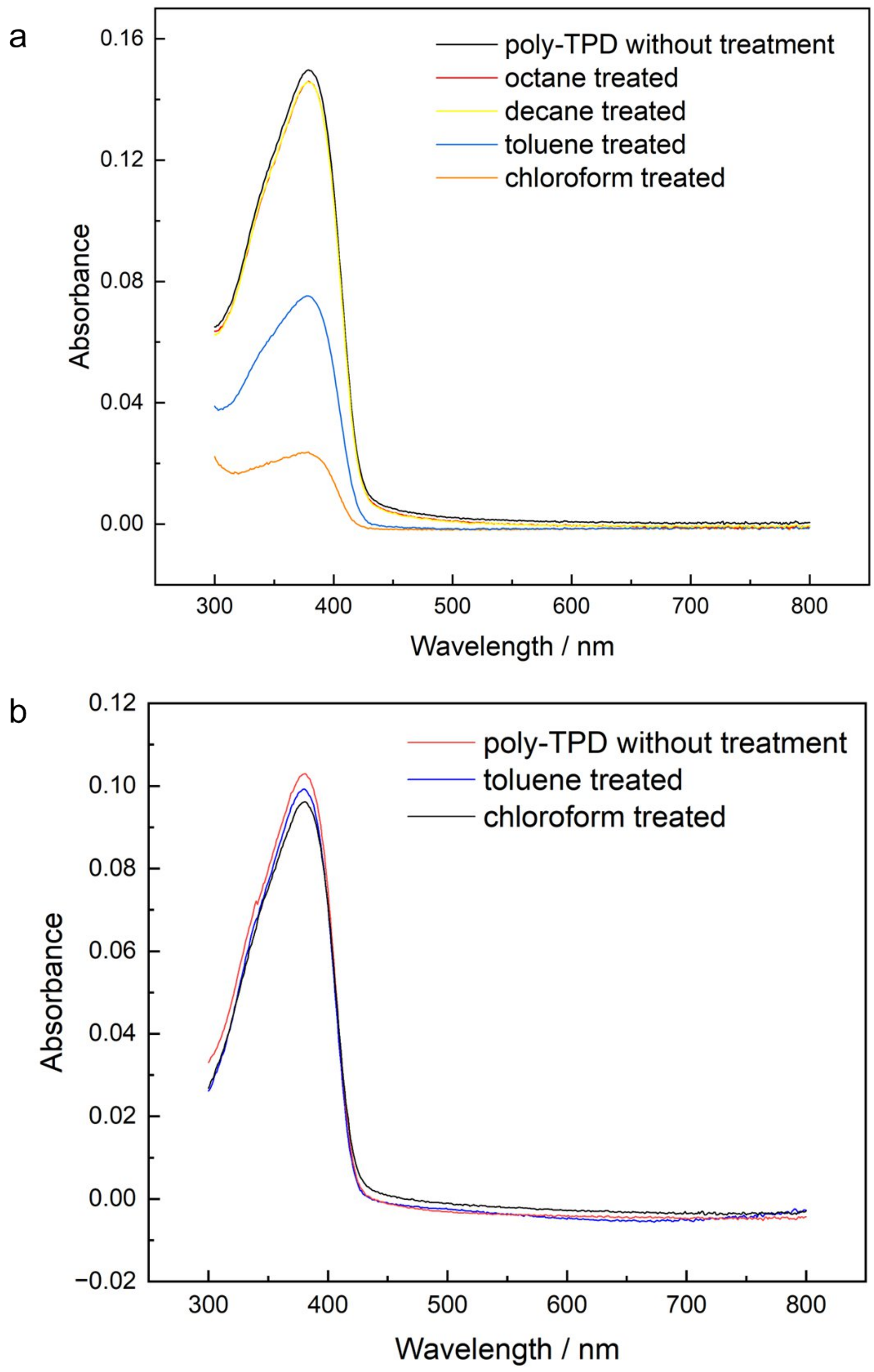

Figure S14. UV–vis absorption spectra of poly-TPD (hole transport layer, HTL) film on PEDOT:PSS film before and after spin-coating using each solvent. (a) Spectra obtained after spin-coating. The data indicate that the thicknesses of poly-TPD films after spin-coating using octane and decane were the same, whereas those after spin-coating with chloroform and toluene were seven- and two-times thinner, respectively. (b) Spectra of poly-TPD films on PEDOT:PSS after spin-coating using each toluene and chloroform. In these cases, the poly-TPD were prepared at concentrations two and seven times higher than the original concentrations, respectively. Therefore, after spin-coating using each solvent, the films had almost same absorbance as before.

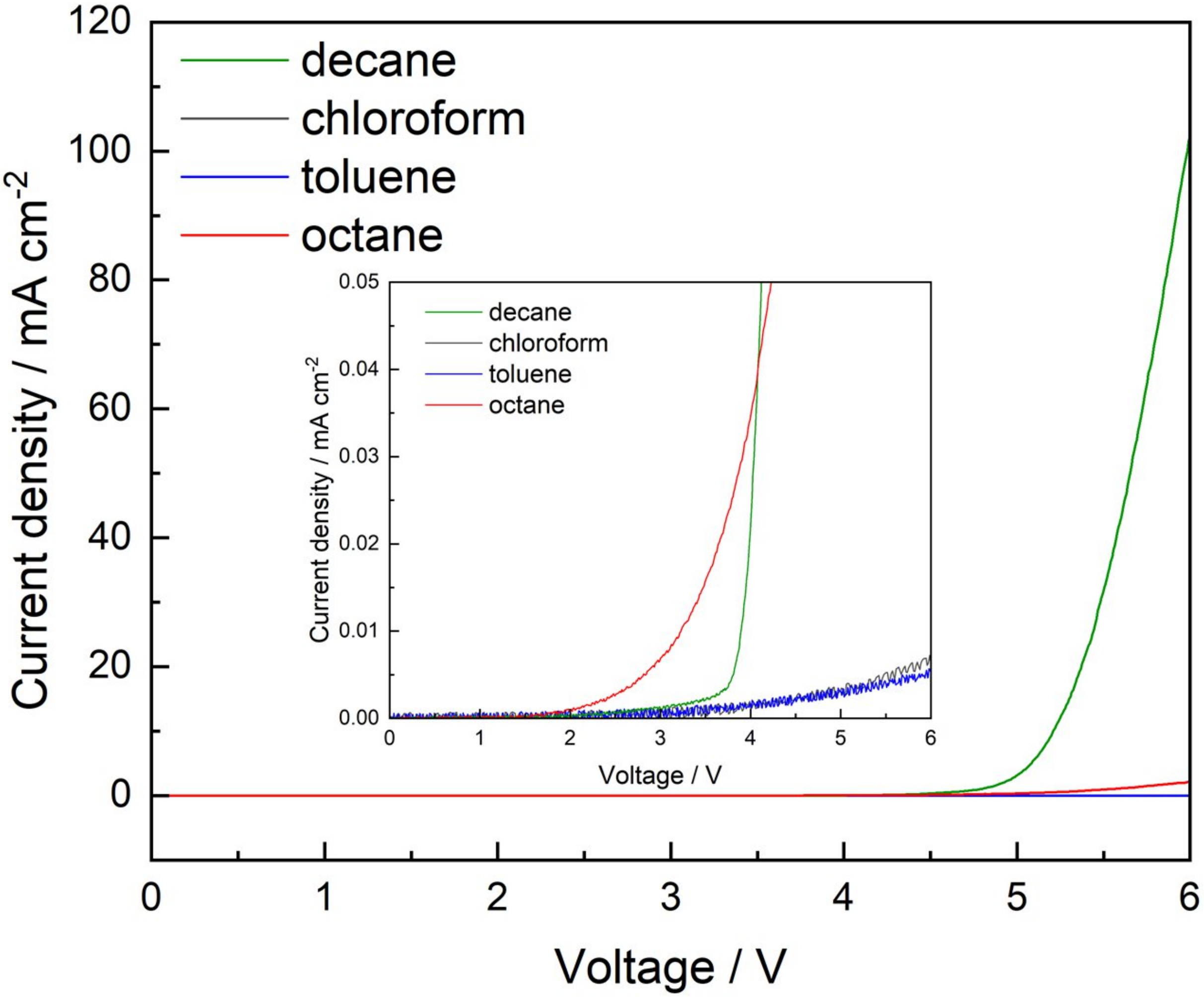

Figure S15. *I–V* curves of the four SiQD LEDs modified to have the same HTL thickness via dissolution of the HTL layer after spin coating using each SiQD dispersion. Inset panel was expanded data in the vertical axis. The performances of the four thus-prepared SiQD LEDs with the same HTL thicknesses were evaluated by measuring the *I–V* curves; the diode profiles of the octane- and decane-dispersed SiQD LEDs are good, but those of the chloroform- and toluene-dispersed SiQD LEDs are poor.

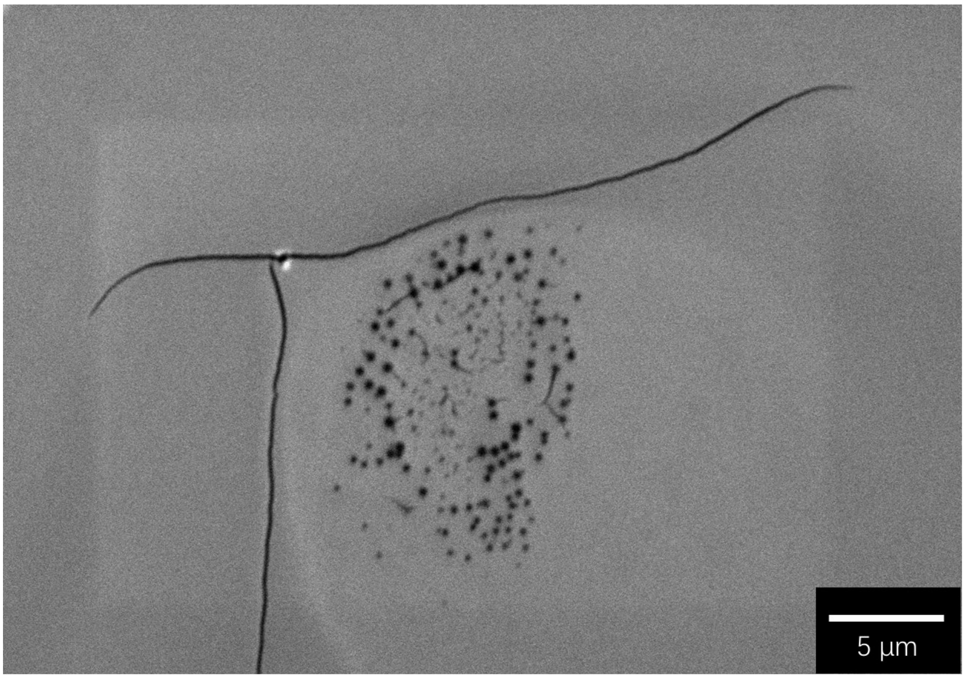

Figure S16. SEM image of ZnO layer of decane-dispersed SiQD LED. Many pinholes can be seen in the layer.

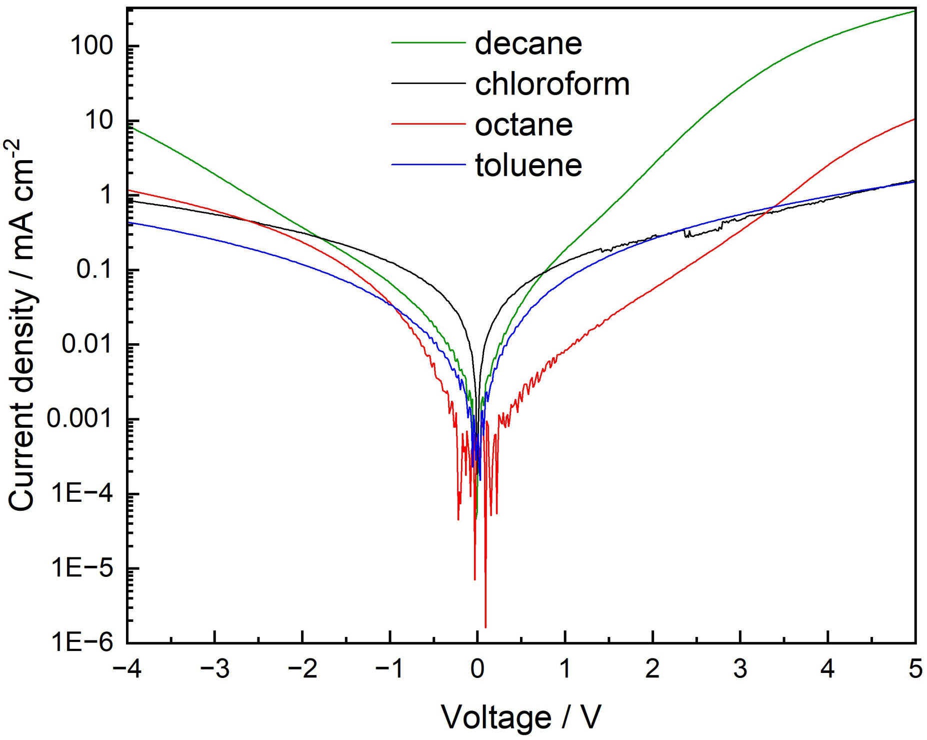

Figure S17. *I–V* curves of the four SiQD LEDs to evaluate leakage currents. The decane-dispersed SiQD LEDs showed the highest leakage current, as observed in the inverse voltage region at −4 V. This was attributed to the fact that these devices had the greatest SiQD aggregate area, resulting in pinholes in the SiQD LED layers that increased the leakage current. In contrast, other three SiQD LEDs showed 10 times lower leakage currents, and the leakage ratio, the current density at −4V divided by that at +4V, can be seen to be the lowest for the octane-based SiQD LED in the three SiQD LEDs.

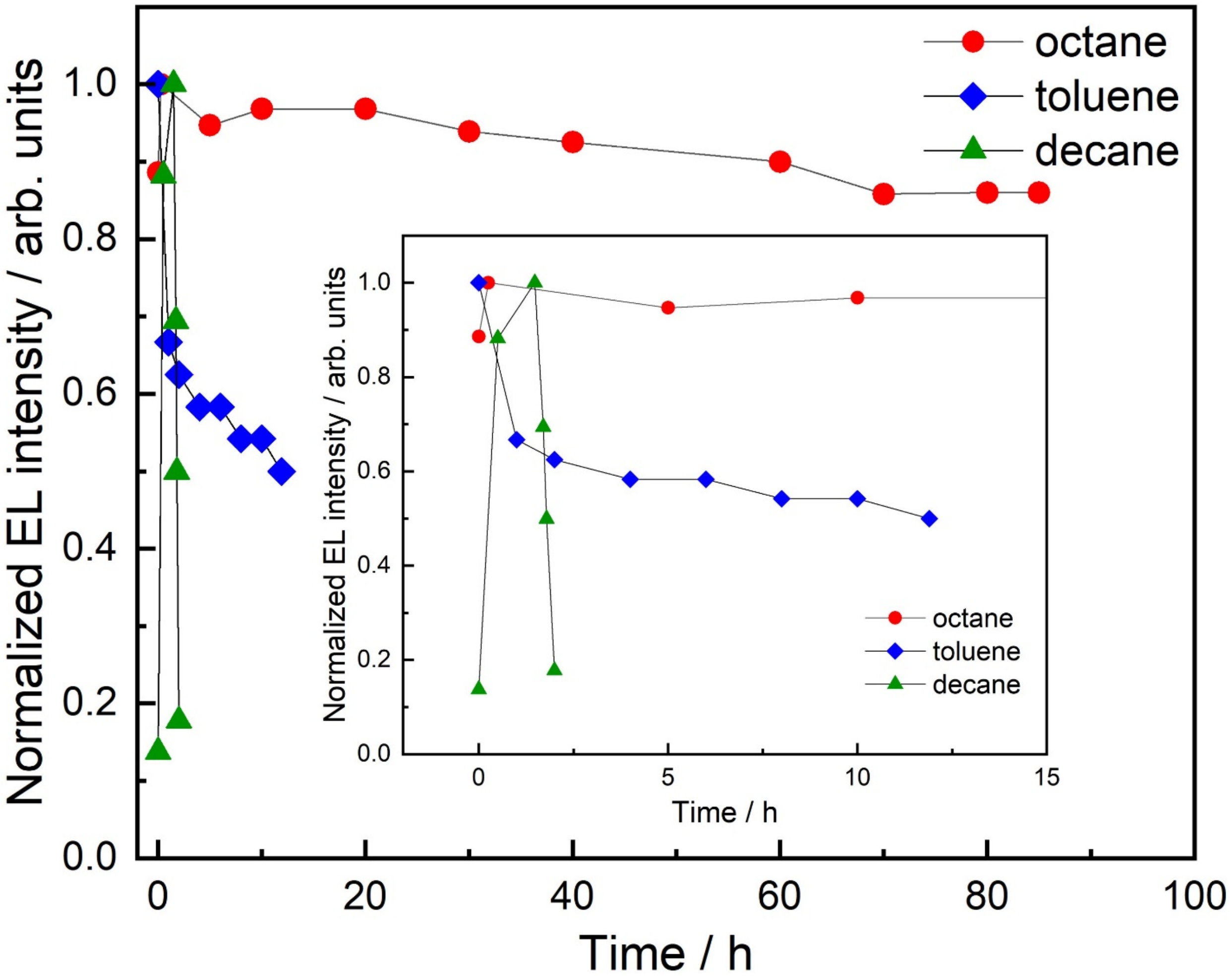

Figure S18. Repeat SiQD LED operation lifetime measurements. The EL intensities of the decane-, octane-, and toluene-dispersed SiQD LEDs are plotted as functions of the device operation time; the data were collected at applied voltages of 4, 6, and 6 V, respectively. The inset shows the same data plotted using a different range for the horizontal axis. In each case, the conditions for the lifetime measurements were as follows: 23 °C temperature, 50% relative humidity, air atmosphere, and the EL intensity was measured in a dark box. The decane-based SiQD LED, which has a high current density, was degraded very quickly at 6 V, preventing accurate lifetime measurements, and hence for this device the lifetime measurements were conducted at the reduced applied voltage of 4 V.

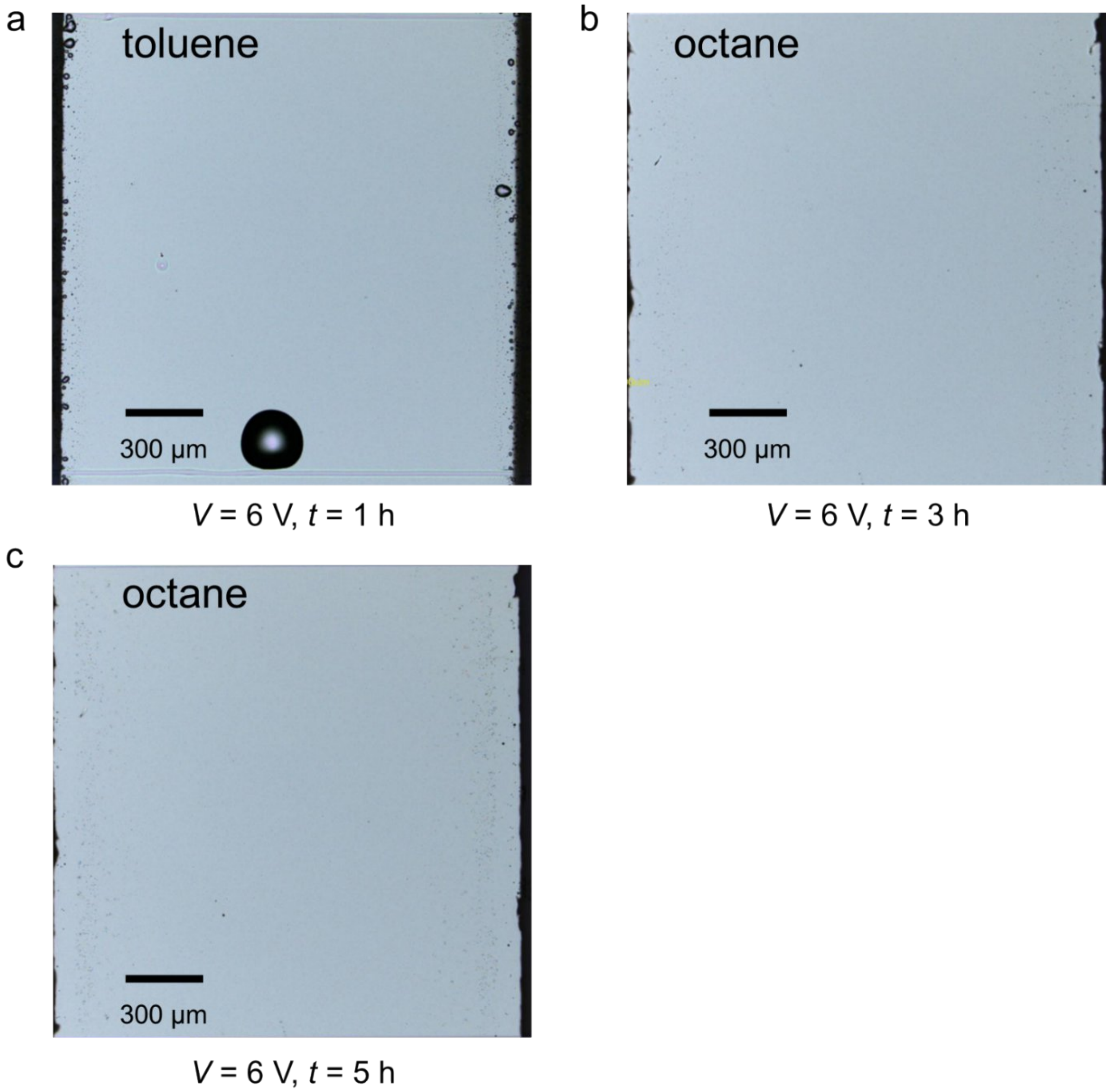

Figure S19. Expanded views of images shown in Figure 6b. Optical micrographs of a) toluene-dispersed SiQD LED after 1 h of operation, b) octane-dispersed SiQD LED after 3 h of operation, and c) octane-dispersed SiQD LED after 5 h of operation. The voltage applied during operation was 6 V in each case. The reason why the results shown in Figure 6b and Figure S19 were obtained under the same voltage is that this allowed relatively easy experimental control, facilitating accurate measurement. In contrast, controlling the current density and luminance during experiments was more difficult. Moreover, adjusting the conditions to obtain a similar current density and/or luminance took time, resulting a period of time in which data collection was not possible. Therefore, the measurements were conducted under the same voltage to set an accurate start time for the measurement series.

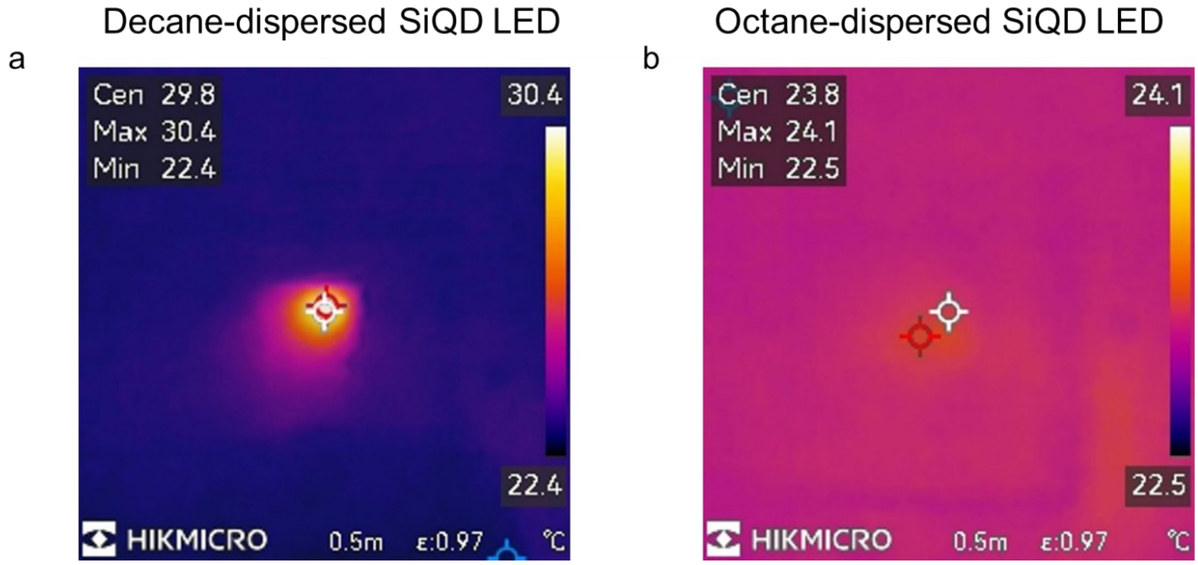

Figure S20. Temperature distribution images of two SiQD LEDs during device operation. The temperatures of the 2 mm × 2 mm active central areas to which the voltage was applied were measured. The maximum temperatures of the (a) decane-and (b) octane-dispersed SiQD LEDs were observed as 30.4 and 24.1 °C, respectively. Both the images were collected within 1 min of the application of 6 V. The current densities of the former and latter LEDs were measured as 95 and 0.736 mA cm^{-2} at 6 V, respectively. The images were captured using a radiation thermometer (HIKMICRO Eco, HangZhou Microimage Software Co., Ltd) in a room temperature environment (23 ℃) with a relative humidity of 32%.

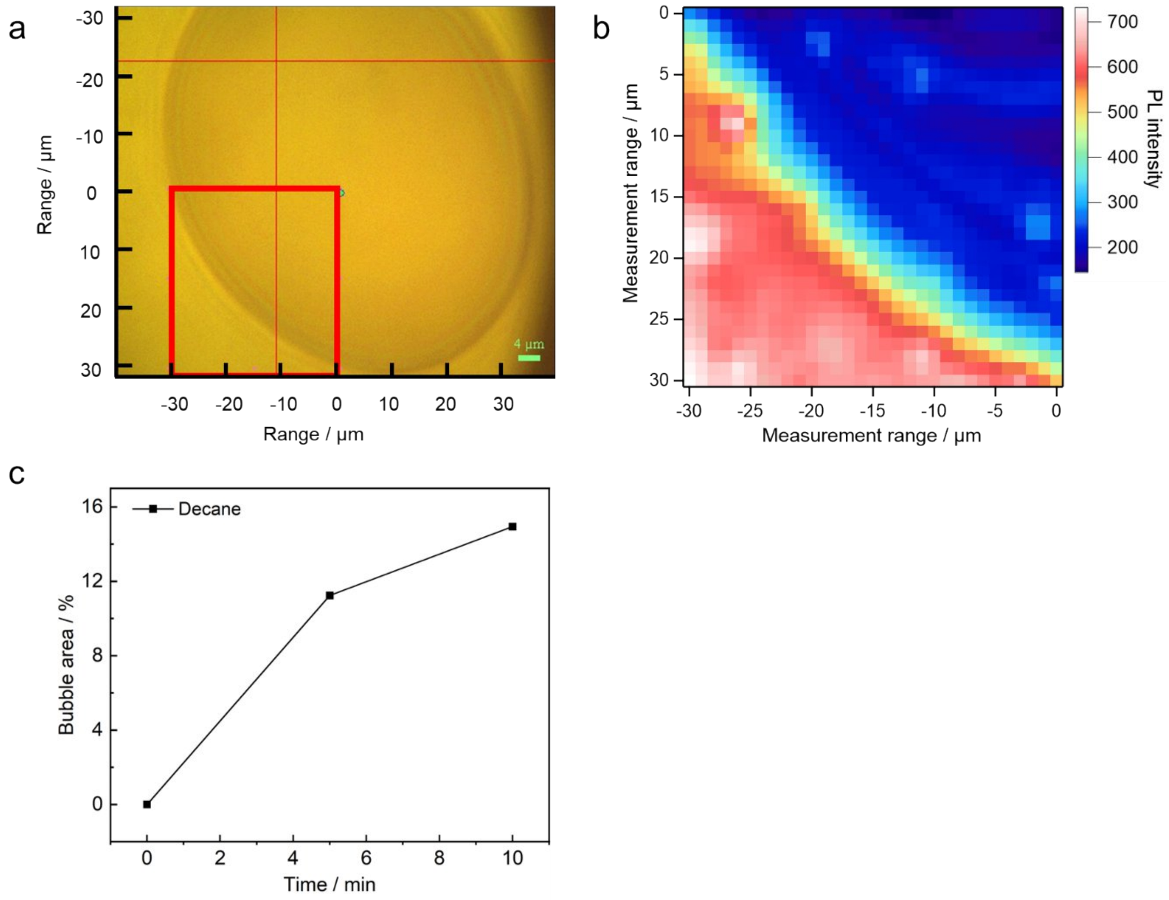

Figure S21. Analysis of microbubbles formed in SiQD LED after voltage application. a) Optical micrograph of a microbubble. b) PL map of the portion of the microbubble outlined by the red rectangle in panel a. c) Bubble area as a percentage of the total SiQD LED area versus device operation time; this data corresponds to that shown in Figure 6b for the decane-dispersed SiQD LED. The bubble area (%) was obtained as the sum of the area of each microbubble divided by the active area (2 mm × 2 mm). The area of each microbubble was calculated by assuming that it was a circle, using its radius as measured in the micrograph. The data were acquired under a constant applied voltage of 6 V.

Table S1. PLQYs of chloroform-, decane-, octane-, and toluene-dispersed SiQDs.

SiQD dispersant:	chloroform	decane	octane	toluene
Excitation wavelength	330 nm	330 nm	330 nm	350 nm
PLQY	63%	46%	43%	51%

Note: the excitation wavelength was set to 350 nm for the toluene-dispersed SiQDs to avoid the absorption band of the solvent (toluene).

Table S2. SiQD device structures, external quantum efficiencies (EQEs), and luminance data reported in the literature

Year	Device structure	λ_{EL} (nm)	Maximum EQE (%)	Maximum luminance (cd m^{-2})$^{a)}$	Maximum OPD$^{b)}$ (μW cm^{-2})	Ref.
2010	ITO/PEDOT:PSS/MEHPPV/SiQD/BCP/LiF/Al	868	0.6	…	85 (12 V)	[16]
2011	ITO/PEDOT:PSS/poly-TPD/SiQD/TiO_2/Al	618	…	…	0.07 (20 V)	[45]
2011	ITO/PEDOT:PSS/poly-TPD/SiQD/Alq_3/LiF/Al	853	8.6	…	130 (16 V)	[10]
2011	ITO/PVK/SiQD/TPBi/Al	685	0.7	58 (30 V, 2000 A m^{-2})	…	[13]
2013	ITO/PEDOT:PSS/poly-TPD/SiQD/TPBi/LiF/Al	680	1.1	22.6 (10 V, 250 A m^{-2})	…	[12]
2015	ITO/PEDOT:PSS/poly-TPD/SiQD/Alq_3/Al	410	…	…	0.7 (6 V)	[5]
2016	Al/MoO_3/TAPC/SiQD/ZnO/PEI/ITO (inverted structure)	690	2.7	…	110 (18 V)	[9]
2017	ITO/MoO_3/PEDOT:PSS/poly-TPD/SiQD/ZnO/Ag	740	2.4	…	19 (16 V)	[46]
2018	Al/MoO_3/CBP/SiQD/ZnO/ITO (inverted structure)	720	3.1	…	…	[25]
2018	ITO/PEDOT:PSS/poly-TPD/SiQD/ZnO/Ca/Al	694	0.018	16.5 (5.4 V, 4000 A m^{-2})	…	[15]
2018	ITO/PEDOT:PSS/poly-TPD/PVK/SiQD/ZnO/Ag	735	6.2	…	640 (9.9 V)	[14]
2018	ITO/PEDOT:PSS/SiQD/TPBi/Al	700	0.3	5.4 (1.4 V, 0.002 A m^{-2})	…	[11]
2020	Al/MoO_3/CBP/SiQD/ZnO/ITO (inverted structure)	755	3.36	…	…	[7]
2021	ITO/PEDOT:PSS/poly-TPD/SiQD/ZnO/Ag	700	1.48	…	734 (6 V)	[47]
2022	Al/MoO_3/CBP/SiQD/ZnO/ITO (inverted structure)	680	2.2	…	8 (12 V)	[4]
2022	ITO/PEDOT:PSS/poly-TPD/SiQD/Alq_3/Al	730	1.92	…	35 (10 V)	[3]
2022	Al/MoO_3/CBP/SiQD/ZnO/ITO (inverted structure)	730	12.2	…	8.16 (5 V)	[8]
2023	ITO/PEDOT:PSS/poly-TPD/SiQD/ZnO/Ag	735	1	…	600 (8 V)	[48]
2024	Al/MoO_3/CBP/SiQD/ZnO/ITO (inverted structure)	730	10.2	…	325 (12 V)	[1]
2024	ITO/PEDOT:PSS/TFB/SiQDs/ZnMgO/Ag	760	13.2	…	480 (4 V)	[49]
2025	ITO/PEDOT:PSS/poly-TPD/SiQD/ZnO/Al	750	16.5	66 (6 V, 930 A m^{-2})$^{c)}$	248 (6 V)$^{c)}$	This study

$^{a)}$Values in parentheses are applied voltages and current densities corresponding to the luminance values; $^{b)}$OPD: optical power density. Values in parentheses are applied voltages corresponding to the measured OPD values. $^{c)}$This luminance was obtained from the data points of Figure S4 a_1 and b_1 of the decane-dispersed SiQD LED (i.e., details in Section S5).

Table S3. SiQD device structures and luminance values obtained from experiments and calculations

Year	Device structure	λ_{EL} (nm)	Luminance (cd m^{-2})$^{a)}$			Ref.
			Experimental value	Value calcd. using Equation S1	Value calcd. using Equation S2	
2011	ITO/PVK/SiQD/TPBi/Al	685	58 (30 V, 2,000 A m^{-2})	…	…	[13]
2013	ITO/PEDOT:PSS/Poly-TPD/SiQD/TPBi/LiF/Al	680	22.6 (10 V, 250 A m^{-2})	…	…	[12]
2018	Al/MoO_3/CBP/SiQD/ZnO/ITO (inverted structure)	720	…	5,000 (5 V, 40 A m^{-2})	10.46	[25]
2018	ITO/PEDOT:PSS/Poly-TPD/SiQD/ZnO/Ca/Al	694	16.5 (5.4 V, 4,000 A m^{-2})	…	…	[15]
2018	ITO/PEDOT:PSS/SiQD/TPBi/Al	700	5.4 (1.4 V, 0.002 A m^{-2})	…	…	[11]
2020	Al/MoO_3/CBP/SiQD/ZnO/ITO (inverted structure)	755	…	19,934 (9 V, 9 A m^{-2})	6.62 (9 V, 9 A m^{-2})	[7]
2025	ITO/PEDOT:PSS/Poly-TPD/SiQD/ZnO/Al	750	14.4 (5.3 V, 24 A m^{-2})	32,158 (5.3 V, 24 A m^{-2})	12.1 (5.3 V, 24 A m^{-2})	This study

$^{a)}$Values in parentheses are applied voltages and current densities corresponding to the luminance values. The values calculated using Equations S1 and S2 are the incorrect (overestimated) and correct values, respectively.

S7. References

[1] H. Ueda, K. Saitow, *ACS Appl. Mater. Interfaces* **2024**, *16*, 985.
[2] S. Terada, H. Ueda, T. Ono, K. Saitow, *ACS Sustain. Chem. Eng.* **2022**, *10*, 1765.
[3] Y. Xu, S. Terada, Y. Xin, H. Ueda, K. Saitow, *ACS Appl. Nano Mater.* **2022**, *5*, 7787.
[4] T. Ono, Y. Xu, T. Sakata, K. Saitow, *ACS Appl. Mater. Interfaces* **2022**, *14*, 1373.
[5] Y. Xin, K. Nishio, K. Saitow, *Appl. Phys. Lett.* **2015**, *106*, 201102.
[6] S. Terada, Y. Xin, K. Saitow, *Chem. Mater.* **2020**, *32*, 8382.
[7] H. Yamada, N. Saitoh, B. Ghosh, Y. Masuda, N. Yoshizawa, N. Shirahata, *J. Phys. Chem. C* **2020**, *124*, 23333.
[8] H. Yamada, J. Watanabe, K. Nemoto, H.-T. Sun, N. Shirahata, *Nanomaterials* **2022**, *12*, 4314.
[9] L. Yao, T. Yu, L. Ba, H. Meng, X. Fang, Y. Wang, L. Li, X. Rong, S. Wang, X. Wang, G. Ran, X. Pi, G. Qin, *J. Mater. Chem. C* **2016**, *4*, 673.
[10] K.-Y. Cheng, R. Anthony, U. R. Kortshagen, R. J. Holmes, *Nano Lett.* **2011**, *11*, 1952.
[11] B. Ghosh, T. Hamaoka, Y. Nemoto, M. Takeguchi, N. Shirahata, *J. Phys. Chem. C* **2018**, *122*, 6422.
[12] F. Maier-Flaig, J. Rinck, M. Stephan, T. Bocksrocker, M. Bruns, C. Kübel, A. K. Powell, G. A. Ozin, U. Lemmer, *Nano Lett.* **2013**, *13*, 475.
[13] D. P. Puzzo, E. J. Henderson, M. G. Helander, Z. Wang, G. A. Ozin, Z. Lu, *Nano Lett.* **2011**, *11*, 1585.
[14] X. Liu, S. Zhao, W. Gu, Y. Zhang, X. Qiao, Z. Ni, X. Pi, D. Yang, *ACS Appl. Mater. Interfaces* 2018, *10*, 5959.
[15] A. Angı, M. Loch, R. Sinelnikov, J. G. C. Veinot, M. Becherer, P. Lugli, B. Rieger, *Nanoscale* **2018**, *10*, 10337.
[16] K.-Y. Cheng, R. Anthony, U. R. Kortshagen, R. J. Holmes, *Nano Lett.* **2010**, *10*, 1154.
[17] V. I. Klimov, A. A. Mikhailovsky, D. W. McBranch, C. A. Leatherdale, M. G. Bawendi, *Science* **2000**, *287*, 1011.
[18] J. Tang, M. Sakamoto, H. Ohta, K. Saitow, *Nanoscale* **2020**, *12*, 4352.
[19] D. Kajiya, K. Saitow, *Nanoscale* **2015**, *7*, 15780.
[20] D. Kajiya, K. Saitow, *ACS Appl. Energy Mater.* **2018**, *1*, 6881.
[21] T. Sakata, D. Kajiya, K. Saitow, *ACS Appl. Mater. Interfaces* **2020**, *12*, 46598.
[22] T. Sakata, K. Saitow, *J. Phys. Chem. Lett.* **2022**, *13*, 653.
[23] M. Sakamoto, S. Terada, T. Mizutani, K. Saitow, *ACS Appl. Mater. Interfaces* **2021**, *13*, 1105.
[24] M. Sakamoto, K. Saitow, *Nanoscale* **2018**, *10*, 22215.
[25] B. Ghosh, H. Yamada, S. Chinnathambi, İ. N. G. Özbilgin, N. Shirahata, *J. Phys. Chem. Lett.* **2018**, *9*, 5400.
[26] V. C. Wood, All inorganic colloidal quantum dot LEDs. Thesis, Massachusetts Institute of Technology, **2007**.
[27] X. Zhu, X. Luo, Y. Deng, H. Wei, Feng Peng, L. Ying, F. Huang, Y. Hu, Y. Jin, *Sci. Adv.* **2024**, *10*, eado0614.
[28] J. Song, O. Wang, H. Shen, Q. Lin, Z. Li, L. Wang, X. Zhang, L. S. Li, *Adv. Funct. Mater.* **2019**, *29*, 1808377.
[29] W. Lu, B.-X. Mi, M. C. W. Chan, Z. Hui, C.-M. Che, N. Zhu, S.-T. Lee, *J. Am. Chem. Soc.* **2004**, *126*, 4958.
[30] M. Shukla, N. Brahme, R. S. Kher, M. S. K. Khokhar, *Indian J. Pure Appl. Phys.* **2011**, *49*, 142.
[31] S. Okamoto, K. Tanaka, Y. Izumi, H. Adachi, T. Yamaji, T. Suzuki, *Jpn. J. Appl. Phys.* **2001**, *40*, L783.

[32] S. Wang, Y. Liu, C. Liu, W. Shao, C. Wang, M. Xiao, G. Chen, Z. Yu, C. Tao, W. Ke, G. Fang, *Laser Photonics Rev.* **2023**, *17*, 2200871.
[33] L. Protesescu, S. Yakunin, S. Kumar, J. Bär, F. Bertolotti, N. Masciocchi, A. Guagliardi, M. Grotevent, I. Shorubalko, M. I. Bodnarchuk, C.-J. Shih, M. V. Kovalenko, *ACS Nano* **2017**, *11*, 3119.
[34] G. Li, F. W. R. Rivarola, N. J. L. K. Davis, S. Bai, T. C. Jellicoe, F. de la Peña, S. Hou, C. Ducati, F. Gao, R. H. Friend, N. C. Greenham, Z.-K. Tan, *Adv. Mater.* **2016**, *28*, 3528.
[35] Q. Van Le, H. W. Jang, S. Y. Kim, *Small Methods* **2018**, *2*, 1700419.
[36] C.-S. Yang, R. A. Bley, S. M. Kauzlarich, H. W. H. Lee, G. R. Delgado, *J. Am. Chem. Soc.* **1999**, *121*, 5191.
[37] M. p. Stewart, E. g. Robins, T. w. Geders, M. j. Allen, H. Cheul Choi, J. m. Buriak, *Phys. Status Solidi A* **2000**, *182*, 109.
[38] C. M. Hessel, M. R. Rasch, J. L. Hueso, B. W. Goodfellow, V. A. Akhavan, P. Puvanakrishnan, J. W. Tunnel, B. A. Korgel, *Small* **2010**, *6*, 2026.
[39] Y. V. Ryabchikov, S. A. Alekseev, V. Lysenko, G. Bremond, J.-M. Bluet, *J. Nanoparticle Res.* **2013**, *15*, 1535.
[40] A. Grill, D. A. Neumayer, *J. Appl. Phys.* **2003**, *94*, 6697.
[41] T. Kaneko, D. Nemoto, A. Horiguchi, N. Miyakawa, *J. Cryst. Growth* **2005**, *275*, e1097.
[42] M. Künle, T. Kaltenbach, P. Löper, A. Hartel, S. Janz, O. Eibl, K.-G. Nickel, *Thin Solid Films* **2010**, *519*, 151.
[43] Y. Lin, T. Y. Tsui, J. J. Vlassak, *J. Electrochem. Soc.* **2006**, *153*, F144.
[44] International Commission on Illumination (CIE), *CIE spectral luminous efficiency for photopic vision*, International Commission on Illumination, https://cie.co.at/detatable/cie-spectral-luminous-efficiency-photopic-vision
[45] C.-C. Tu, L. Tang, J. Huang, A. Voutsas, L. Y. Lin, *Appl. Phys. Lett.* **2011**, *98*, 213102.
[46] W. Gu, X. Liu, X. Pi, X. Dai, S. Zhao, L. Yao, D. Li, Y. Jin, M. Xu, D. Yang, G. Qin, *IEEE Photonics J.* **2017**, *9*, 1.
[47] J. Mock, E. Groß, M. J. Kloberg, B. Rieger, M. Becherer, *Adv. Photonics Res.* **2021**, *2*, 2100083.
[48] I. T. Cheong, J. Mock, M. Kallergi, E. Groß, A. Meldrum, B. Rieger, M. Becherer, J. G. C. Veinot, *Adv. Opt. Mater.* **2023**, *11*, 2201834.
[49] Q. He, K. Wang, D. Li, D. Yang, X. Pi, *Adv. Opt. Mater.* **2024**, *12*, 2302422.
